# Development of (polyvinyl alcohol/*O*-carboxymethyl chitosan/PbO) nanocomposites for efficient gamma radiation protection

**DOI:** 10.1038/s41598-026-64674-7

**Published:** 2026-08-08

**Authors:** Ahmed M. Elkhatib, Hammed H. A. M. Hassan, Basma A. Abdelmgeed, Mahmoud I. Abbas, Mona M. Gouda

**Affiliations:** 1 Physics Department, Faculty of Science, Alexandria University, Alexandria, 21511 Egypt; 2 Chemistry Department, Faculty of Science, Alexandria University, P. O. Box 2, Moharram Beck, Alexandria, 21568 Egypt

**Keywords:** Carboxymethyl chitosan, Nanocomposite, TEM, Permeability, γ-Radiation shielding, Chemistry, Materials science, Nanoscience and technology, Physics

## Abstract

A polymeric blend based on polyvinyl alcohol (PVA) / carboxymethyl chitosan was developed for gamma radiation shielding applications. The nanocomposite films were fabricated using a simple solution casting method, where the readily available nanosized lead oxide PbO particles were dispersed within a polymer matrix composed of PVA, polyvinylpyrrolidone (PVP), and citric acid. The dispersion process was assisted by sonication before crosslinking, ensuring uniform distribution of oxide particles throughout the films. The physicochemical properties of the synthesized nanocomposites were systematically characterized using scanning electron microscopy (SEM), transmission electron microscopy (TEM), energy-dispersive X-ray spectroscopy (EDX), X-ray diffraction (XRD), ultraviolet–visible spectroscopy (UV–Vis spectroscopy), thermogravimetric analysis (TGA), differential scanning calorimetry (DSC), water vapor permeability (WVP), soil burial degradation, and total soluble matter (water absorption) measurements. Gamma shielding performance was evaluated through the determination of linear and mass attenuation coefficients (μ and μ/ρ) at photon energies ranging from 59.53 to 1332 keV, using a NaI (Tl) scintillation detector with standard gamma-ray sources (Am-241, Ba-133, Eu-152, Cs-137, and Co-60). Additional shielding parameters, including half-value layer (HVL), tenth-value layer (TVL), and mean free path (MFP), were calculated. The experimental linear and mass attenuation coefficients for the blank sample, which closely matched the theoretical predictions from XCOM, verified the produced films’ outstanding gamma radiation shielding capabilities. PbO nanoparticles were incorporated into the chitosan matrix to produce a uniform and compact film structure. HVL, TVL, and MFP, among other computed shielding factors, showed how well the nanocomposites attenuated gamma photons throughout a broad energy range. Furthermore, the structural stability, uniform shape, and environmental degradability of the composites were validated by physicochemical studies. These findings demonstrate that carboxymethyl chitosan/PbO nanocomposite films are attractive options for long-term radiation protection, as they combine efficient radiation-shielding capabilities with other desirable properties. While carboxymethyl chitosan/PbO nanocomposite films show promising radiation shielding efficiency, their long-term stability remains a concern, as partial material degradation may affect their sustained performance.

## Introduction

Radiative particles such as alpha, beta, and neutron particles, along with electromagnetic wave emissions, are commonly produced as by-products in various industrial sectors, including nuclear power plants, the medical field, and space exploration. The electromagnetic (EM) spectrum encompasses a wide range of non-ionizing and ionizing radiations, with frequencies ranging from 10^2^ to 10^22^ Hz and corresponding wavelengths from 10^8^ to 10⁻^12^ meters. However, unintentional or sudden exposure to these forms of radiation can lead to serious biological effects, including carcinogenesis, genetic mutations, DNA damage, chromosomal abnormalities, blood cell impairment, and failure of vital organs. Therefore, there is a constant and growing need to develop advanced radiation shielding materials that offer effective protection against these hazardous exposures^1^. Gamma rays possess high penetration capabilities, allowing them to pass through the human body and induce ionization processes that can damage biological tissues. Consequently, safeguarding individuals, equipment, and the surrounding environment from radiation exposure is critical to minimizing potential health and structural risks. As a result, radiation protection has emerged as a fundamental area of research in the field of physics. To mitigate external radiation hazards, three core principles are applied: minimizing exposure time, increasing the distance from the radiation source, and implementing effective shielding. Among these, the selection of appropriate shielding materials plays a pivotal role. Accordingly, recent scientific efforts have been increasingly directed toward the development and optimization of advanced materials with superior radiation attenuation properties^2^. Lead is widely recognized as one of the most effective conventional materials for shielding against radiation exposure. Alongside lead, concrete remains a commonly employed shielding material, particularly in radiation therapy centers and nuclear reactor environments^3^. Additionally, several high-density metals such as tungsten, bismuth, copper, and steel have demonstrated strong potential as effective radiation barriers. In recent years, there has been growing interest in the development of alternative shielding solutions involving composite materials^4^. These include polymers, glasses, metal alloys, and specialized mortars integrated with micro- or nanoparticles as fillers^5,6^. Such composites are being actively explored due to their promising shielding capabilities, as well as their adaptability to specific functional and structural requirements across various radiation protection applications^1,2^.

Over the past century, the use of plastics has increased dramatically due to the world’s expanding population. It was anticipated that the chemical industry would create plastics that reduce environmental pollution from synthetic polymers in the last century due to the poor rate of degradability of plastics, which is regarded as a serious issue^7^. Using polymer blends and composites to develop more environmentally compatible materials is an important strategy for future research. These days, plastics made from petrochemicals are being replaced with biodegradable polymer mixes and composites. Tests were conducted on totally or partially biodegradable films using natural biopolymers such as chitosan, cellulose, and starch, either by themselves or in combination with synthetic polymers. The well-known and plentiful natural polymer chitosan is generated from the exoskeleton of crustaceans. It has many hydroxy and amino groups and is categorized as a natural polymer due to the presence of the degradable enzyme chitosanase^8^.

In the context of growing environmental concerns and the urgent need for sustainable alternatives to conventional radiation shielding materials, the development of biodegradable composites has become a vital area of research. Chitosan, a natural linear polysaccharide derived from the partial deacetylation of chitin, is gaining prominence in the development of environmentally friendly composite materials^9^. Chitosan is derived from renewable sources, such as shrimp and crab shells, and possesses several favorable properties: it is biocompatible, biodegradable, non-toxic, and capable of forming stable films and hydrogels^10^. These attributes make it an excellent matrix for incorporating functional fillers to create advanced materials suitable for radiation shielding^11^. Chemically, chitosan contains free amino groups (–NH₂) that enable ionic interactions, crosslinking, and blending with other polymers or inorganic additives, enhancing its physicochemical versatility^10^.

In this study, we developed a novel composite based on chitosan, reinforced with polyvinyl alcohol (PVA) and polyvinylpyrrolidone (PVP), to improve film stability, and processability. To impart radiation protection capabilities, lead oxide (PbO) was introduced due to its high atomic number and density, which allow for efficient attenuation of gamma. The incorporation of PbO into the biopolymeric matrix forms a hybrid structure with potential applications in personal protective equipment, temporary shielding layers in medical imaging rooms, or radiation packaging. This aligns with current global efforts to reduce non-degradable polymer waste and replace hazardous materials with safer alternatives^8^. We note that PbO is a toxic heavy metal oxide; therefore, while the polymer matrix in our composites is biodegradable, these materials are not entirely green, instead, they represent an approach that pairs a more environmentally compatible polymer matrix with high-Z fillers to improve shielding while recognizing that Pb-containing materials require careful handling and end-of-life management. Additional functional compounds were added to enhance the composite’s structural cohesion and prolong its functionality under radiation exposure while preserving the partial biodegradability of the polymer phase.

This work highlights the potential of chitosan-based composites as dual-function materials capable of shielding against harmful radiation while also offering environmental compatibility. The strategy combines polymer chemistry and materials science to develop a composite with promising shielding performance, although the presence of PbO requires careful consideration of safety and environmental impacts. Also, the structural and functional properties of the synthesized polymer nanocomposite were thoroughly investigated using various analytical techniques, including SEM, TEM, EDX, XRD, UV, water vapor permeability testing, soil burial degradation analysis, total soluble matter (water absorption), and thermal analysis. The gamma radiation shielding performance of the polymer nanocomposite was assessed by examining its absorption and attenuation capabilities. We also underscore those further assessments (leaching tests, abrasion/wear testing, and life-cycle analysis) are required to quantify possible Pb release and to define safe application scenarios; such tests are proposed as follow-up work.

## Materials and methods

### Materials

PVA (M.W., 14000, BDH Chemicals Ltd, Poole, England), PVP (M.W., 40000, Techno Pharmachem, India), chitosan (M.W., >700kDa, DD 82.4%, Alpha Chemicals, India), chloroacetic acid (CDH Ltd, India), citric acid (Adwic, Pharmaceutical Chemicals Co., Egypt), hydrated lead acetate (Merck), lead oxide (PbO) nanoparticles were synthesized following a standard procedure^12^, double-distilled water, solvents such as EtOH, MeOH, Et_2_O, EtOAc, Py (Al-Gomhorya Chemicals Co., Egypt), and analytical reagent-grade NaCl, NaOH, NaHCO_3_, Na_2_SO_4,_ and CaCl_2_ (Al-Gomhorya Chemicals Co., Egypt). PbO nano powder was handled in a fume hood using appropriate personal protective equipment (gloves, lab coat, respirator as required), and all waste containing Pb was collected for hazardous-waste disposal according to institutional guidelines.

### Preparation of composite film

- *Synthesis of O-carboxymethyl chitosan (OCC) 2*

To improve film-forming properties and moisture resistance, modification of poly(vinyl alcohol) to its carboxymethylated PVA derivative was essential. Chitosan (10 g) powder was soaked in aqueous NaOH solution (13.5 g dissolved in 200 mL H_2_O) for 3h at rt, then filtered. The pre-treated chitosan **1** was dissolved in isopropanol/water (80:20 v/v) and stirred for 1h. A second solution of chloroacetic acid (15g) dissolved in isopropyl alcohol (20 ml) was prepared and dropwise added over 10 min to the chitosan solution. The obtained solution was cooled in an ice bath and stirred for a further 5h and then kept frozen for 24 h and filtered by washing with 70 % ethanol. The desired powder was dried at 60 °C for 12 h^13^.

- *Fabrication of crosslinked PVA/PVP/OCC composite film using citric acid 3*

The PVA solution was first prepared by dissolving PVA (3 g) in preheated distilled water (30 ml), and stirring was continued at 80°C for 1h. In a second-round flask, PVP (2g) was dissolved in preheated distilled water (30 ml) and stirred at 80 °C for 1h. The PVA/PVP blend was prepared by mixing the two polymers, and stirring continued for 1h at 80 °C to ensure a homogenous blend. To this blend, the OCC **2** solution (prepared by dissolving 1g in 20 ml of water at 80 °C), followed by the citric acid solution (prepared by dissolving 0.6 g of citric acid in 10 ml of water at 80 °C), was subsequently added. The mixture was stirred at 80 °C for 2 h and at rt for an additional 2 h to ensure homogeneity. The resulting solution was cast onto a dried Petri dish (12 cm width) and allowed to dry at rt, forming a thin film 3 after six days. The thickness of the specimen was about 0.5 mm.

- *Fabrication of crosslinked PVA/PVP/OCC/citric acid composite films 4 containing different concentrations of the nanosized PdO (General method)*

Similarly, PVA/PVP/OCC/citric acid/PbO composite films were produced by adding the nanosized PbO (0.4g, 0.8g, 1.2g, and 1.6g) before the cross-linker to the mixture, then uniformly dispersed by sonicating the mixture for 20 min to fabricate films having 7.14%, 13.33%, 18.75%, and 23.53% lead oxide contents, respectively, then worked up as mentioned above. We tested PbO loadings up to 23.53 wt%; higher loadings produced brittle, non-processable films during preliminary trials and were therefore not pursued. Mechanical properties (tensile strength, elongation at break, Young’s modulus) were not measured in this study, and because film embrittlement and handling limits prevented reliable testing at higher loadings, we cannot yet identify the trade-off point between shielding performance and usability. Targeted mechanical characterization and process-optimization experiments are planned for follow-up work to determine the maximum practical PbO content and improve film toughness where needed.

PVP was included to improve blend miscibility and assist the uniform dispersion of PbO. Citric acid served as a crosslinker to improve water resistance and thermal stability. O-carboxymethyl chitosan (OCC) provides biodegradability and additional hydrogen-bonding interactions to enhance matrix cohesion. The polymer ratios were selected after preliminary trials that balanced film formability, flexibility, and processability. The films were cast successfully and could be handled during preparation, but flexibility was not quantified experimentally.

**Fig. 1:**
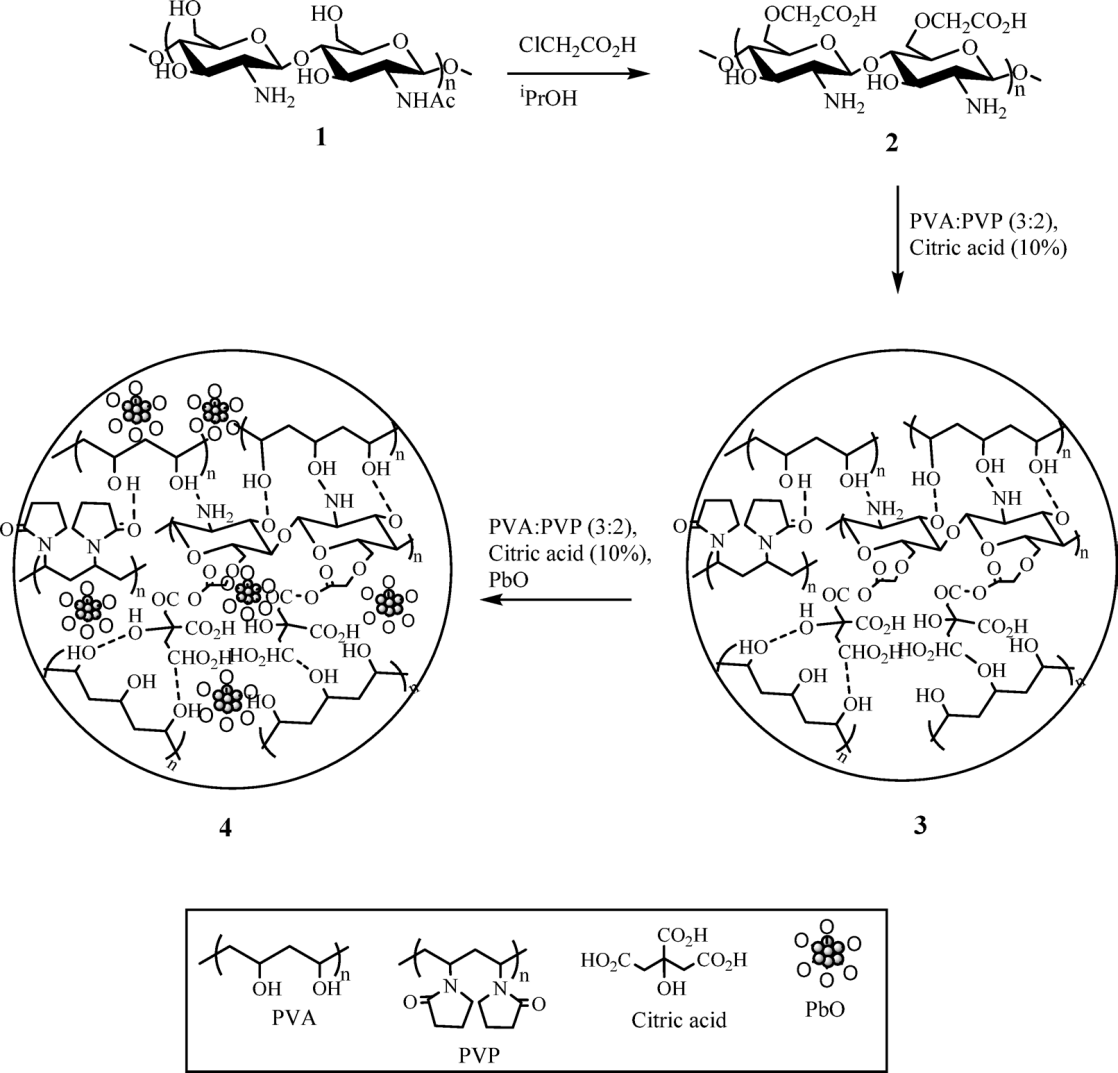
Synthetic pathway to PVA/PVP/*O*-carboxymethyl chitosan/citric acid 3 and PVA/PVP/PVP/O-carboxymethyl chitosan/citric acid/PbO nanocomposites 4.

**Fig. 2:**
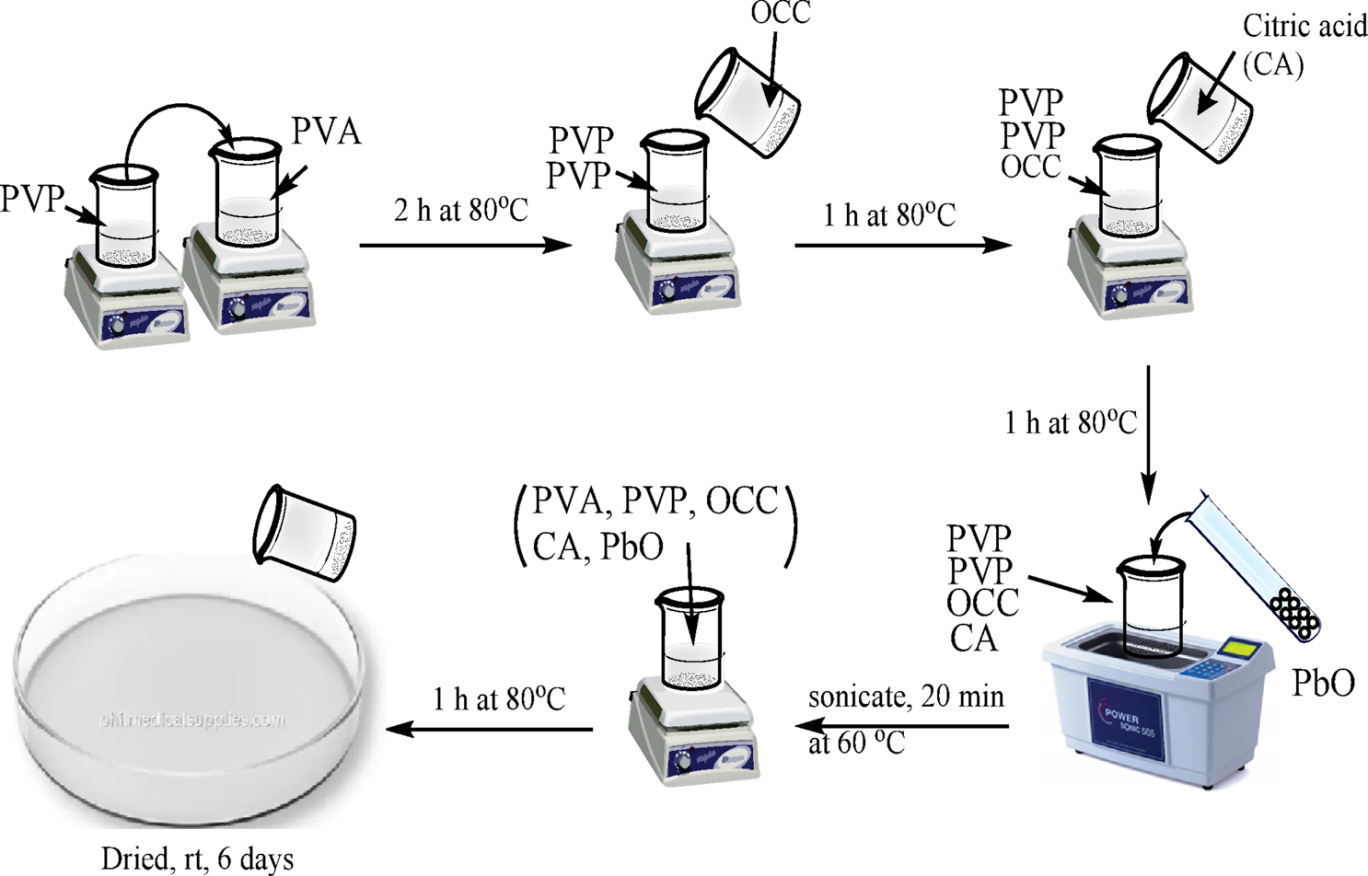
In situ preparation of PVA/PVP/OCC/citric acid/PbO composite film 4.

Figure 1 shows the synthetic pathway to PVA/PVP/O-carboxymethyl chitosan/citric acid **3** and PVA/PVP/O-carboxymethyl chitosan/citric acid/PbO nanocomposites **4**. At the same time, the process of in situ preparation of composites 4 and 5 is depicted in Fig. 2. The samples are labeled as B1 for the composite without PbO, B2 for the composite containing 7.14 % PbO, B3 for the composite containing 13.33 % PbO, B4 for the composite containing 18.75 % PbO, and B5 for the composite containing 23.53 % PbO.

### Characterization techniques

Absorption spectra were measured with a UV 500 UV-Vis spectrometer at room temperature (rt) in DMSO with a polymer concentration of 2 mg/10 mL. Thermogravimetric (TG) and differential thermogravimetric (DTG) analyses were carried out in a stream of nitrogen from 20 to 400 °C using a Shimadzu DTG-60H thermal analyzer. The experimental conditions were a platinum crucible, a nitrogen atmosphere with a flow rate of 30 mL/min, and a heating rate of 10°C/min. Differential scanning calorimetry (DSC-TGA) analyses were carried out using SDT-Q600-V20.5-Build-15 at the microanalytical unit, Cairo University. The morphologies of polymers were observed by Scanning Electron Microscope (SEM) (JEOL-JSMIT 200) at the E-Microscope Unit, Faculty of Science, Alexandria University. The samples were coated using an ion sputtering coating device, then the samples were placed inside the electron microscope with an operating voltage of 20 kV, and the magnification orders were 1000, 5000, and 10,000. TEM was performed for lead oxide (PbO) NPs on a JEOL JEM-2100 high-resolution transmission electron microscope at an accelerating voltage of 20 kV at the Faculty of Science, Alexandria University, Egypt. X-ray diffraction (XRD) measurements were performed at the Central Laboratory, Faculty of Science, Alexandria University, Egypt, using a standard diffractometer to investigate the crystalline structure of the prepared samples.

### Water vapor permeability (WVP)

WVP of the films was determined according to ASTM E96-00 (ASTM, 2000). Briefly, approximately 15 g of silica gel (pre-annealed at 140 °C) was placed into each cup (diameter 4.3 cm, height 4.5 cm). Composite films B1, B2, B3, B4, and B5 were carefully and tightly placed on the open ends of the cups. The cups were placed in a desiccator containing a saturated solution of K_2_SO_4_ in water (98% relative humidity)^14^.

The sample film was cut into a circle of 4.3 cm diameter, and the test area was 14.52 cm^2^. The setup was subjected to a temperature and relative humidity of 38 °C and 90 %, respectively. The determination of water vapor penetration through the film and absorbed by silica gel was carried out by weight change measurements (with accuracy ± 0.0005 g) as a function of time. Water vapor transmission rate (WVTR) was calculated as:

1 $${\mathrm{WVTR}} = {\raise0.5ex\hbox{$\scriptstyle {\mathrm{G}}$} \kern-0.1em/\kern-0.15em \lower0.25ex\hbox{$\scriptstyle {\mathrm{t}}$}} \times {\mathrm{A}}$$

where G is the weight change (g), t is the time (h), and A is the test area (m^2^).

WVP was calculated as:

2 $${\mathrm{WVP}} = {\text{WVPR }} \times {\text{ T}}/{\mathrm{DP}}$$

Where T is the thickness of the test specimen (cm) and DP is the partial pressure difference of the water vapor across the film.

### Total soluble matter (water absorption)

Total soluble matter was measured by immersion in 50 mL of distilled water. The flasks were stored in the environmental chamber at 25 °C for 1h, 2h, 3h, 4h and 5h, respectively. After each immersion step, specimens were air-dried and weighed. Total soluble matter was calculated in relation to dry mass, and it was expressed as the percentage of the film dry matter solubilized.

3 $${\mathrm{Water}}\,{\mathrm{Absorption}}(\% ) = \frac{{W_{{wet}} - W_{{dry}} }}{{W_{{dry}} }} \times 100$$

where ${W}_{wet}$ ​ is the weight of the plastic sample after water immersion, and ${W}_{dry}$ is the weight before immersion.

### Soil burial degradation

The soil burial degradation of the prepared nanocomposite films was tested using a slight modification of the previously reported method^15^. Briefly, the film sample (1.415 × 1.415 cm^2^) was dried at 80 °C and weighed. The dry sample films are buried in black soil at a depth of 5 cm, and 10 mL of water is added every day for up to 6 months. After 6 months of treatment, the buried films were carefully removed, rinsed with water, and dried at 80 °C. The dried weights of the degraded samples were measured, and the percentage degradation was calculated using Eq. (4).

4 $${\mathrm{Soil}}\,{\mathrm{burial}}\,{\mathrm{degradation}}\% = \frac{{{\mathrm{Initial}}\,{\mathrm{wt}} - {\mathrm{Final}}\,{\mathrm{wt}}}}{{{\mathrm{Initial}}\,{\mathrm{wt}}}} \times 100$$

### Setup for gamma-ray spectroscopy

This study measured the levels of gamma radiation using a sodium iodide scintillation detector (NaI). A photomultiplier tube (PMT) and a multichannel analyzer (MCA) were linked to the detector in order to acquire spectral data. Utilizing common radioactive sources (such as Cs-137 and Co-60), the system was calibrated to guarantee precise energy and efficiency calibration. Table 1 lists the gamma-ray energies of the standard sources used^16,17^.

**Table 1:** Standard gamma-ray sources

Nuclide	Gamma-ray energies (keV)
^241^Am	59.53
^137^Cs	661.66
^133^Ba	80.99, 356.01
^60^Co	1173.23, 1332.50
^152^Eu	121.78

**Fig. 3:**
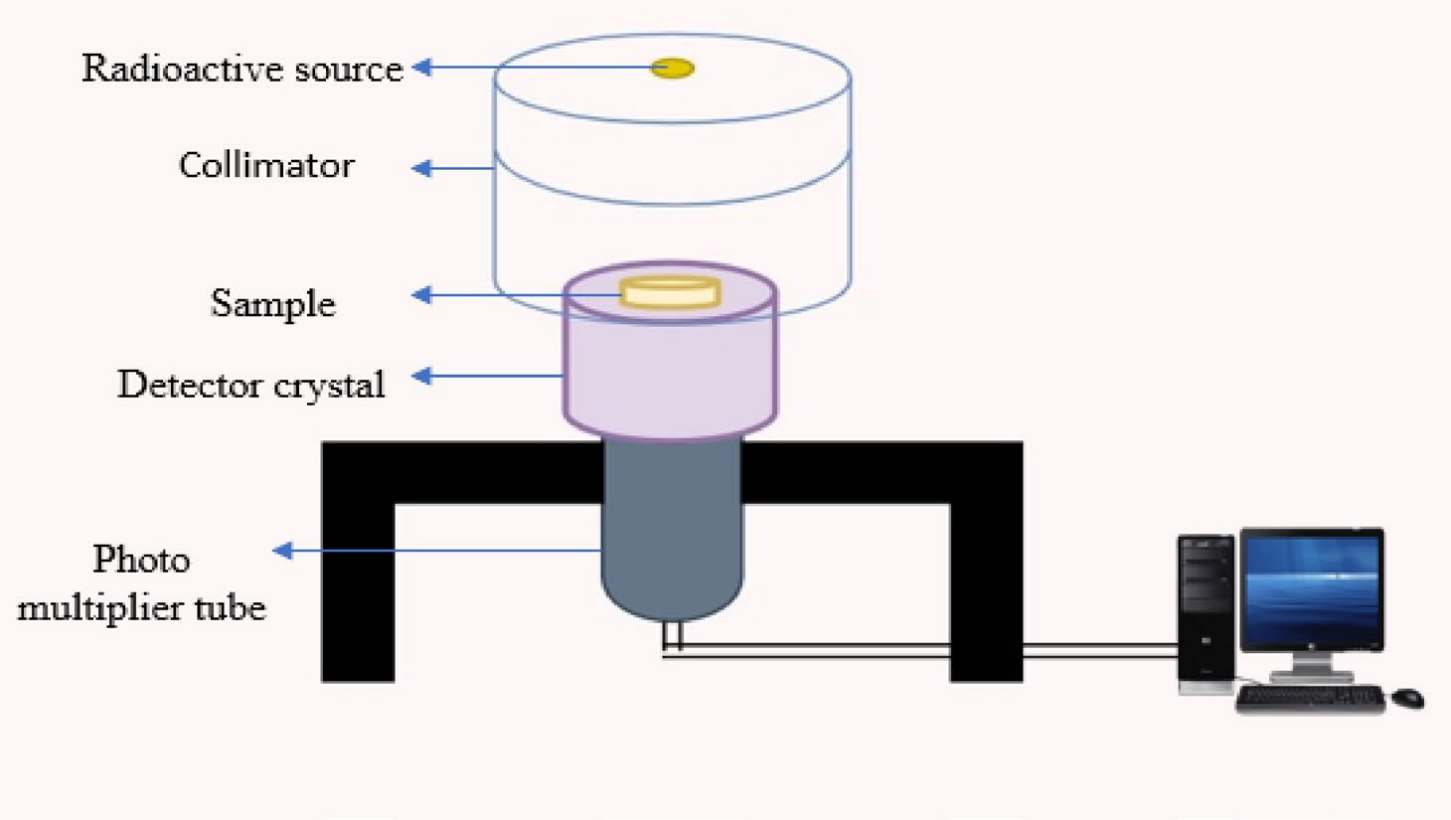
The experimental setup illustrates the positioning of the sample relative to the radioactive source and detector.

A set distance between the radiation source and the detector was used for all measurements to reduce measurement uncertainty and preserve geometric consistency. To guarantee that readings could be compared, this fixed geometry was rigorously maintained throughout all studies^18^. Figure 3 illustrates the configuration of the source–detector setup.

### Radiation parameters

Radiation shielding is based on the principle of attenuation, where the intensity of gamma rays decreases as they pass through a barrier material due to photon absorption and scattering. When gamma radiation passes through an absorber of thickness X, its intensity decreases exponentially according to Beer–Lambert’s law^19^.

5 $${\mathrm{I}} = {\mathrm{I}}_{0} {\mathrm{e}}^{{ - \mu {\mathrm{x}}}}$$

where I_0​_ is the initial intensity of the gamma rays, I is the transmitted intensity after passing through the material, μ is the linear attenuation coefficient (LAC), and X is the thickness of the absorber (in cm).

Mass attenuation coefficient (MAC), which is the linear attenuation coefficient divided by the material density ρ, representing the attenuation per unit mass.

6 $$\mathrm{M}\mathrm{A}\mathrm{C} =\frac{\mu }{\uprho }$$

The half-value layer (HVL) is defined as the material thickness required to reduce gamma-ray intensity to 50% of its initial value and is calculated using the following equation:

7 $$\mathrm{H}\mathrm{V}\mathrm{L} = \frac{\mathrm{L}\mathrm{n}(2)}{\upmu }$$

Tenth-value layer (TVL) refers to the thickness required to reduce the intensity to one-tenth of its original value and is given by the following equation:

8 $$\mathrm{T}\mathrm{V}\mathrm{L} =\frac{\mathrm{L}\mathrm{n}(10)}{\upmu }$$

Mean free path (MFP), which is described by Eq. (9), is the average distance a photon travels inside the material without interaction.

9 $$\mathrm{M}\mathrm{F}\mathrm{P} =\frac{1}{\upmu }$$

## Results and discussion

### Energy dispersive x-ray spectroscopy (EDX)

**Fig. 4:**
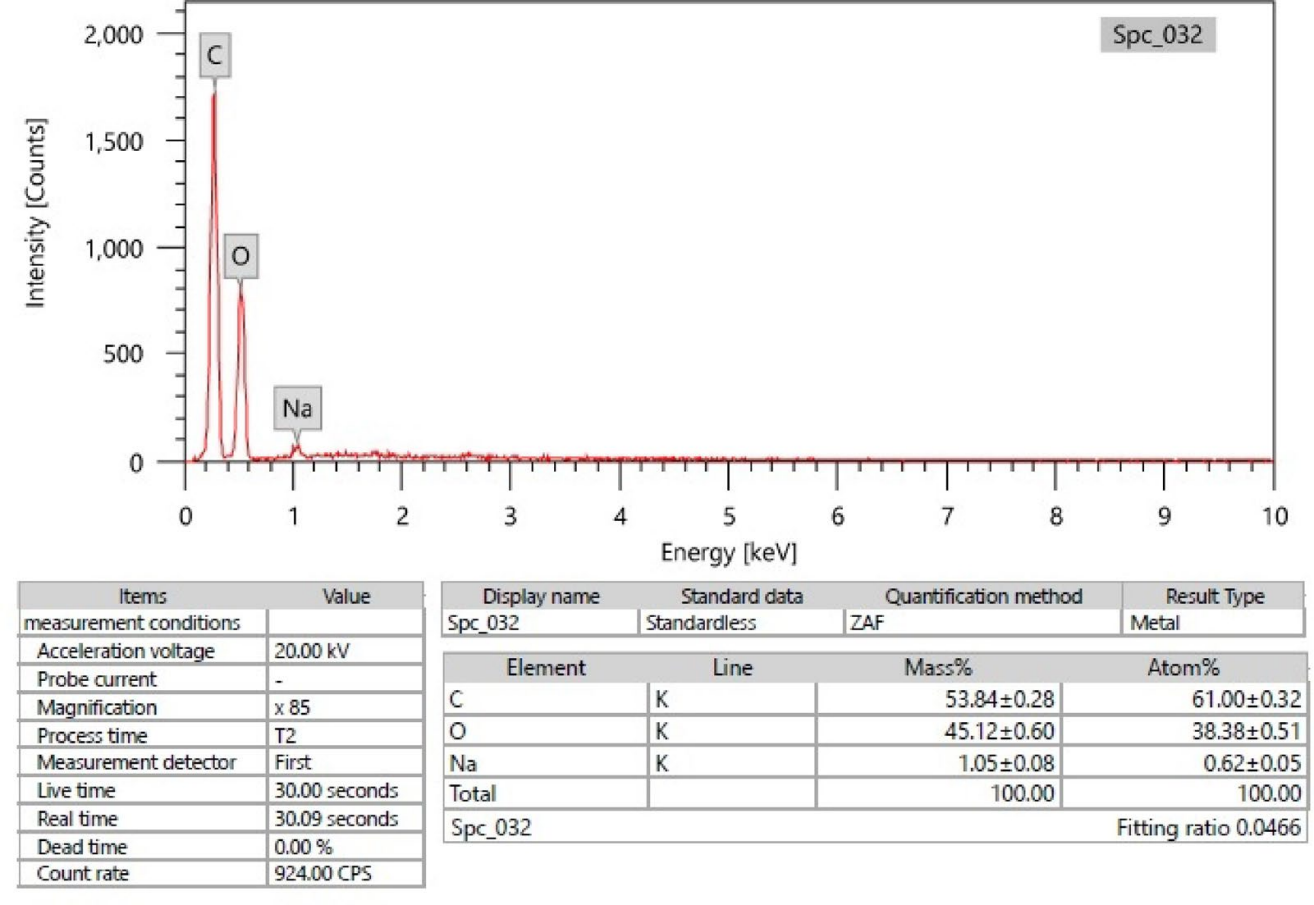
EDX spectrum of sample B1.

**Fig. 5:**
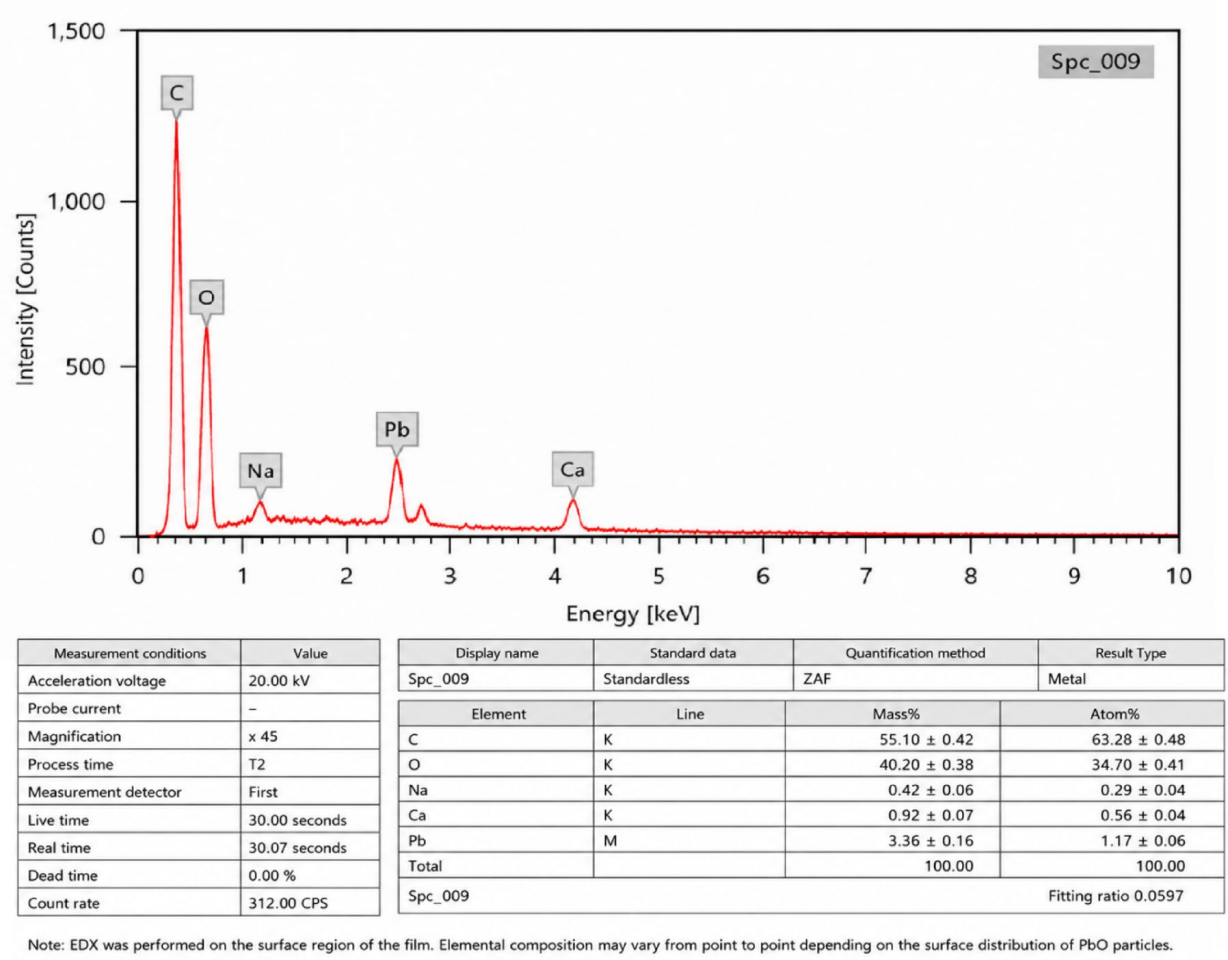
EDX spectrum of sample B5.

Figures 4 and 5 present the energy-dispersive X-ray spectroscopy (EDX) results for reference sample B1, used to determine the elemental composition of the polymer matrix before PbO incorporation, and for sample B5 after PbO addition. EDX analysis was done on a localized surface region, so the measured Pb content is more about that specific surface composition rather than the overall bulk state of the film. The elemental makeup of the neat polymer film, B1, was first found experimentally via EDX analysis. These values were then treated as a kind of reference point when looking at the nanocomposite set (B2–B5), where PbO was included at different levels 7.14, 13.33, 18.75 and 23.53 wt%, respectively. After that, for every sample the elemental compositions were estimated by taking into account the theoretical PbO fraction and then rescaling the total elemental weight percent so it sums to 100%, to keep the whole series consistent. To double check that the filler truly got incorporated, another EDX spectrum was taken for the B5 sample, and it showed a clear Pb signal, which confirms the existence of lead species inside the polymer matrix. Overall, the findings point to a steady, almost monotonic rise of Pb content as the PbO loading increases, which supports compositional tuning and suggests that the inorganic filler is effectively embedded in the polymer system.

### X-ray diffraction

**Fig. 6:**
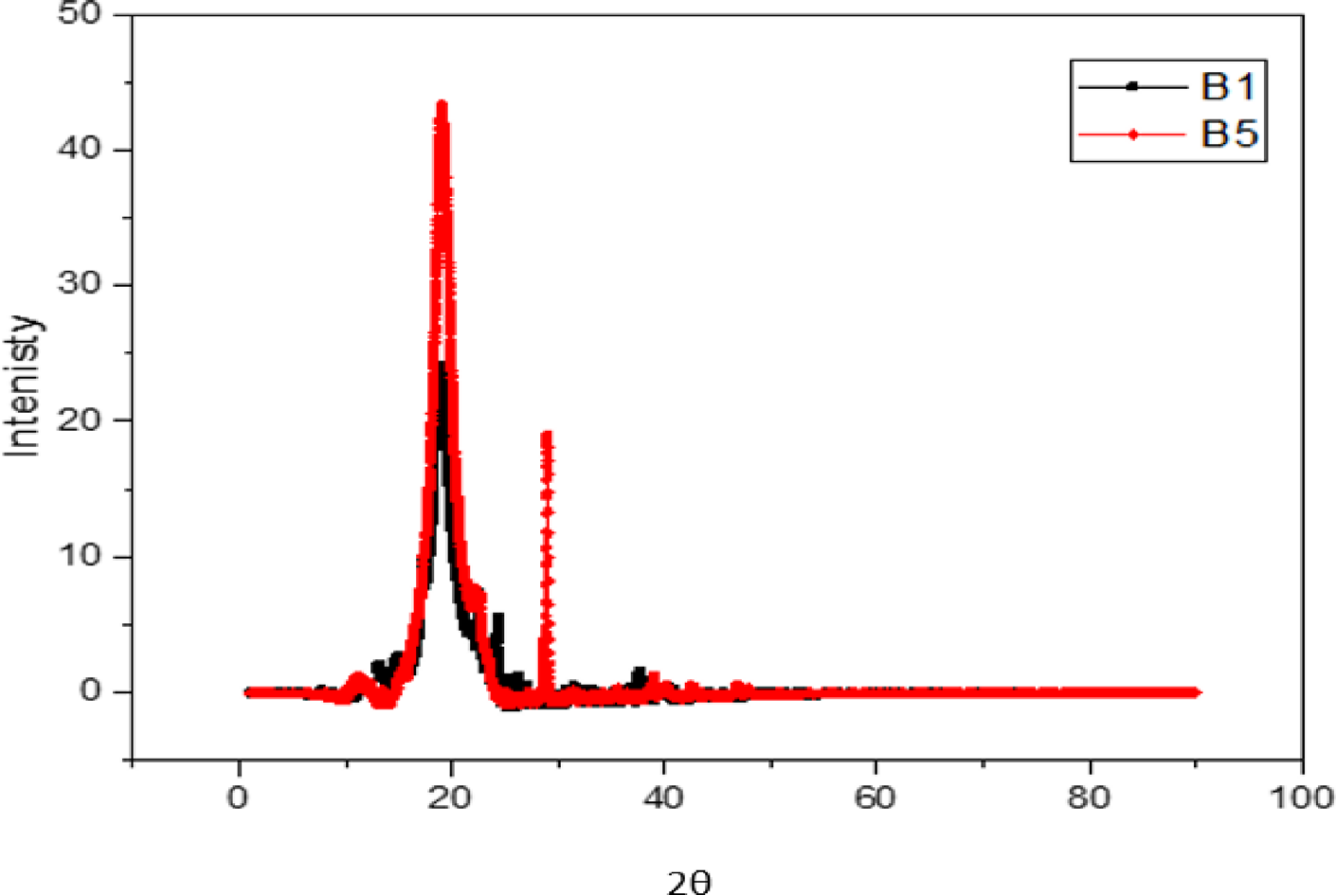
Comparison of XRD spectra for B1 and B5 samples.

The XRD patterns of the reference composite B1 and PbO-reinforced composite B5 are depicted in Fig. 6. The diffraction halo around 2θ ≈ 20° in the B1 pattern confirms the semi-crystalline nature of the polymer matrix. When adding 40 wt.% nano-PbO (B5), a number of sharp diffraction peaks are developed, confirming crystalline PbO. The first peak, with its major intensity at about 2θ ≈ 29°, is consistent with the (101) plane. Other smaller but detectable peaks might correspond to the planes of (002), (110), and (112). These peaks correlate well with the standard diffraction data for tetragonal PbO as recorded in JCPDS card no. 85-0711. With increasing intensity and sharpness, peaks can be recognized, which contribute to the due improvement in crystallinity due to higher loading of PbO and its uniform dispersion within the polymer matrix.

**Table 2:** Soil burial degradation for the free polymer film B1 and nanocomposite samples with varying additive concentrations B2, B3, B4, B5.

Sample	Mass before G	After 6 months g	Deviation %
B1	0.0863	0.0724	16.107
B2	0.0976	0.0894	8.402
B3	0.1020	0.0869	14.804
B4	0.0966	0.0817	15.424
B5	0.1332	0.1135	14.790

### Scanning electron microscopy (SEM) analysis

**Fig. 7:**
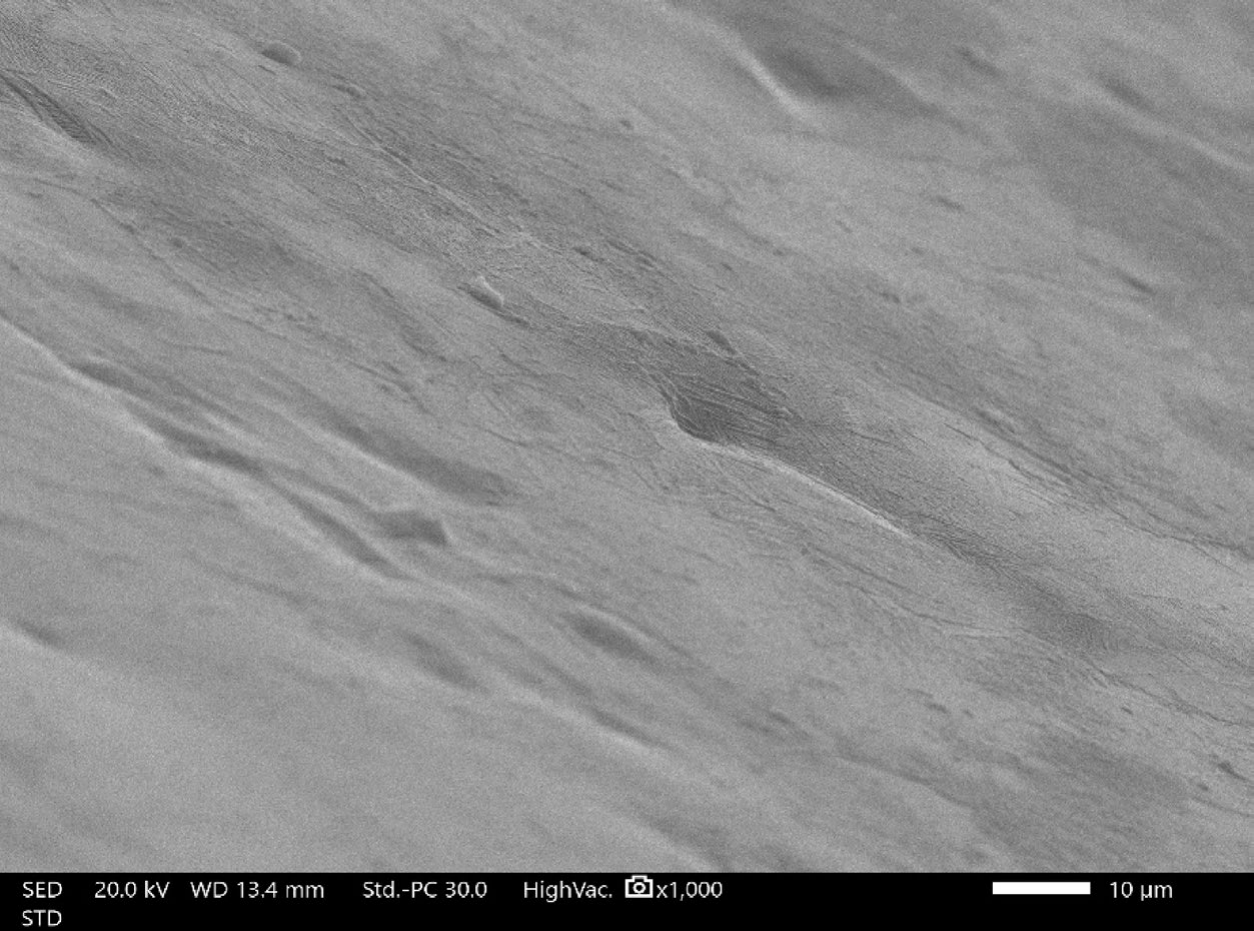
SEM image of the undoped bulk sample (B1) surface showing a relatively homogeneous morphology with no visible agglomerations.

**Fig. 8:**
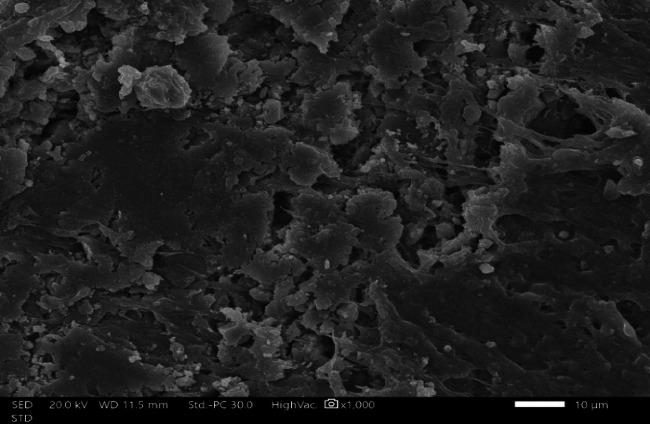
SEM image of the Pb-doped sample (B5) showing surface morphology changes with noticeable grain clustering, indicating the influence of lead doping on the microstructure.

The SEM pictures show how the two polymer samples differ in their morphology. With no discernible inclusions, agglomerates, or phase separation, the lead-free polymer’s surface is smooth and uniform, as shown in Fig. 7. With no inorganic fillers, this homogeneity represents a pure polymeric matrix. While physically stable, the lack of high atomic number elements may restrict its use in radiation shielding. On the other hand, Fig. 8, which corresponds to the polymer sample (B5) loaded with 40 weight percent PbO, exhibits a markedly changed morphology with finely dispersed PbO particles throughout the matrix; the distribution of PbO appears highly uniform, with no evidence of particle agglomeration or interfacial detachment, suggesting strong interaction and compatibility between the inorganic filler and the polymer phase; this well-dispersed microstructure is especially beneficial for radiation shielding applications, as it eliminates localized weaknesses in the material and promotes consistent attenuation behavior. The addition of PbO clearly improved the microstructural features of the composite, transforming it from a homogenous organic phase to a composite material with increased density and atomic number factors that are essential for effective radiation attenuation.

**Fig. 9:**
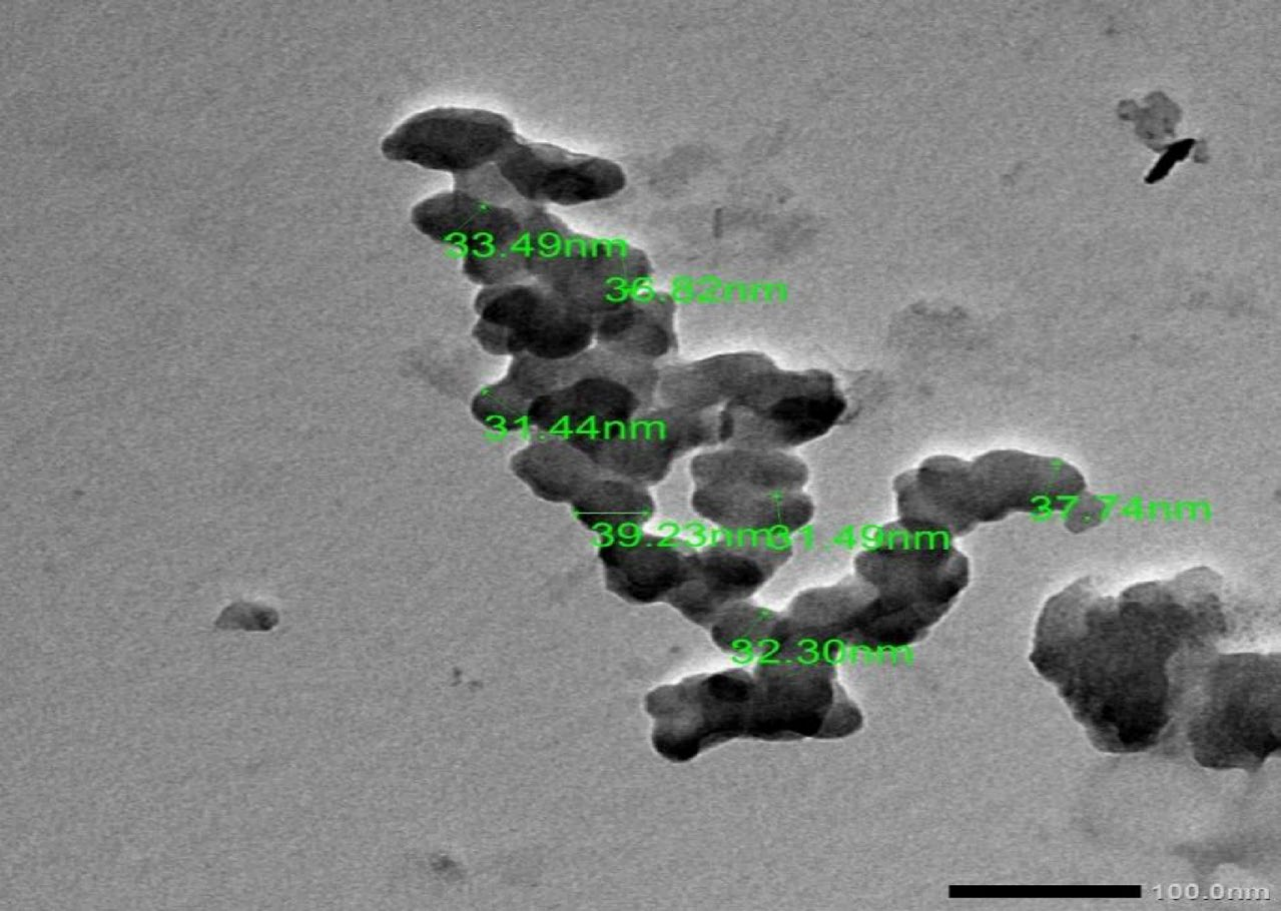
TEM image showing the morphology and particle size distribution of the prepared nanoparticles (~35 nm average).

### Transmission electron microscopy (TEM) analysis

The TEM micrograph displays the synthesized nanoparticles’ morphology. With a distinct propensity to aggregate, the particles have an approximately spherical to irregular shape. The graphic shows the particle size distribution, which has an average size of about 35 nm and ranges roughly from 31 to 39 nm (Fig. 9). This dimension at the nanoscale verifies that the nanoparticles were successfully created. The high surface energy and robust interparticle interactions are responsible for the minor size fluctuations and partial aggregation seen. All things considered, the TEM data show that the particles are nanoscale and are in line with the anticipated structural features.

### Thermal analysis

**Fig. 10:**
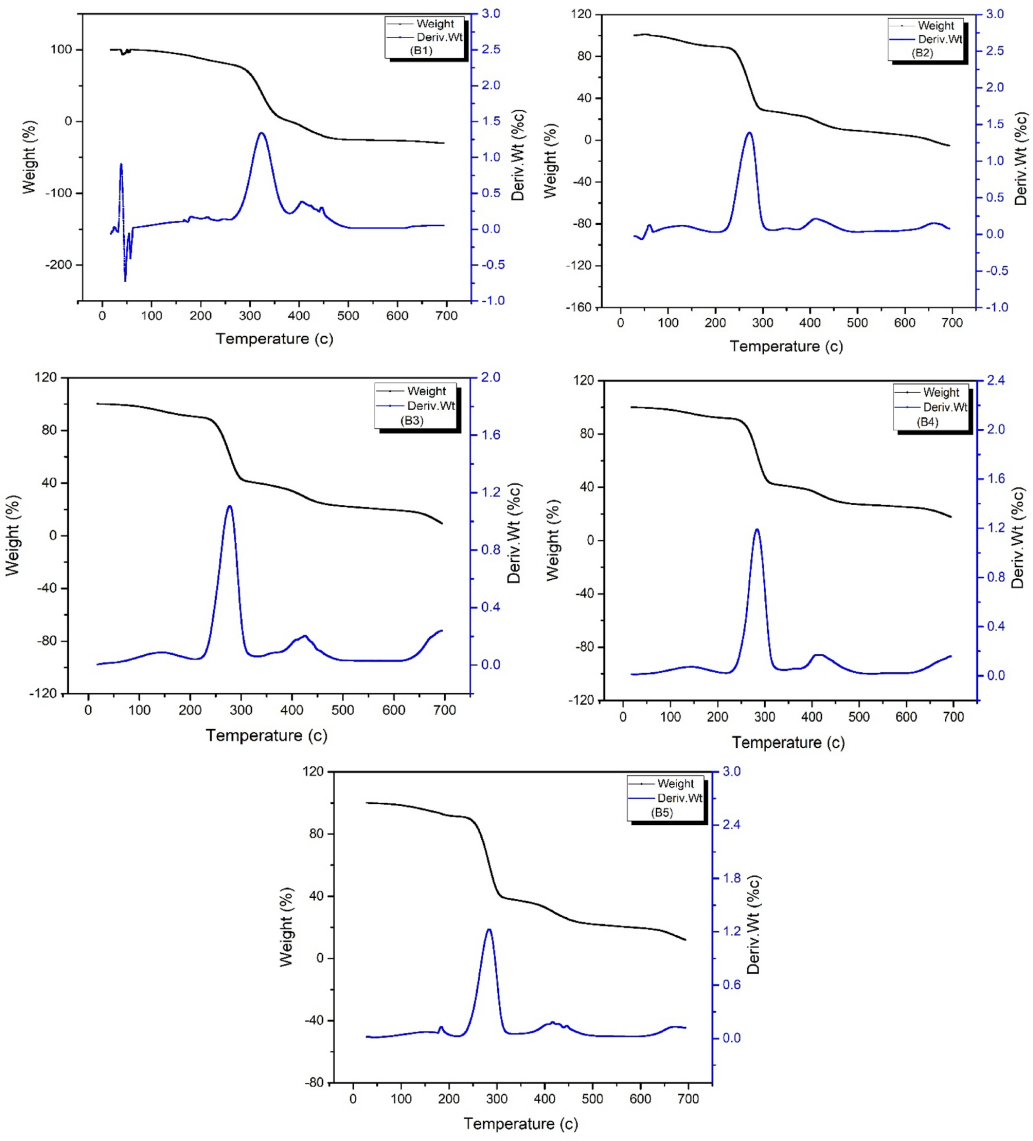
TGA/DTG thermograms of composite films.

**Fig. 11:**
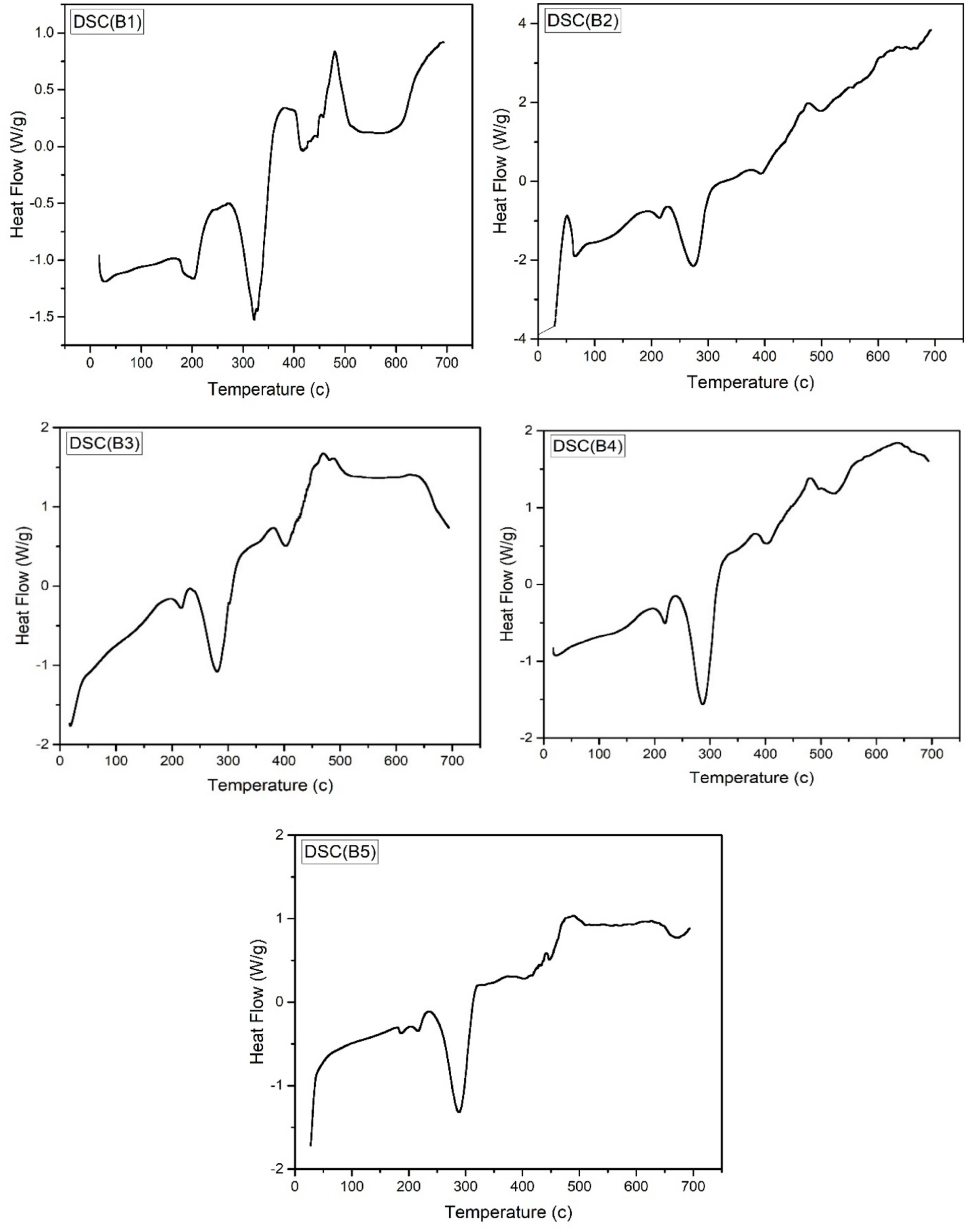
DSC thermograms of composite films.

Thermal stability is characterized by the weight loss of the sample after heating in the temperature range of 25–500 °C. Thermal data of composite films 4 and 5 containing 7.14 %, 13.33%, 18.75%, and 23.53% of PbO were examined utilizing thermogravimetric analysis (TGA), derivative thermogravimetric analysis (DrTGA), and differential thermal analysis (DTA) methods under a nitrogen atmosphere. The TGA/DrTGA profile curves of composite film 4, Fig. 10B1, displayed successive decomposition in three cumulative stages at 241 °C (17.68% wt loss), 401 °C (85.83% wt loss), and 681 °C (22.23% wt loss), leaving at >700 °C a 30.21% value of the sample weight as a residue. The DTG showed a strong main exotherm at 323 °C and two other weak endotherms at 200 °C and 407 °C. The DSC spectrum of 4, Fig. 11B1, exhibited three endothermic peaks at 204 °C (energy 53.68 J/g), 322 °C (energy 345.9 J/g), 412 °C (energy 13.33 J/g), and one exotherm peak at 480 °C (energy 94.59 J/g) due to elimination of side chain substituents and subsequent morphological changes. In particular, the DSC spectrum displays one exothermic degradation peak. This is ascribed to the slow to medium crystallization, and the consequent evolved heat flow over a temperature range was lost in the baseline. The TGA/DrTGA profile curves of the composite containing 7.14% PbO film 5, Fig. 10B2, displayed three decomposition exotherms at 187°C (10.17% wt loss), 345°C (63.73% wt loss), and 684°C (29.28% wt loss). The TGA software reported an apparent final mass value of −5.16 wt% at 693°C. Since a negative residual mass is physically impossible, this value is attributed to baseline drift and instrumental correction effects at high temperatures rather than a real residue. Therefore, the final residual mass can be considered approximately zero within the experimental uncertainty. The DTG showed a weak broad exotherm at 200°C, a strong main exotherm at 271.9°C, and two other endotherms at 411.8 °C and 661.8 °C. The DSC spectrum of 5 (7.14% PbO content), Fig. 11B2, exhibited three endotherms at 64 °C (energy 104.1 J/g), 215 °C (energy 15.83 J/g), 274 (energy 375.0 J/g), and two exotherms at 372 °C (energy 20.92 J/g) and 500 (energy 49.2 J/g). The TGA/DrTGA profile curves of the composite containing 13.33% PbO film 5, Fig. 10B3, displayed three decomposition peaks at 133 °C (4.41% wt loss), 211 °C (4.84% wt loss), 329 °C (49.87% wt loss), and 682 °C (10.11% wt loss), leaving a 9.75% value at 692 °C as residue. The DTG showed a weak broad exotherm at 141.29 °C, a strong main exotherm at 277.7 °C, and a weak endotherm at 425.9 °C. The DSC spectrum of 5 (13.33% PbO content), Fig. 11B3, exhibited three endotherms at 39 °C (energy 22.58 J/g), 216 (energy 16.83 J/g), 281 sharp endotherm (energy 303.7 J/g), an endotherm at 401 C (energy 20.32 J/g), and three additional exotherms at 462 (energy 5.43 J/g), 490C (energy 3.76 J/g), and 642 (energy 55.25 J/g). The TGA/DrTGA profile curves of the composite containing 18.75 % PbO film 5, Fig. 10B4, displayed three decomposition peaks at 207 °C (7.91 % wt loss), 360 °C (51.62 % wt loss), and 684 °C (20.33 % wt loss), leaving a 17.77 % value at 693 °C as residue. The DTG showed a weak broad exotherm at 200 °C, a strong main exotherm at 283.7 °C, and a weak endotherm at 420.9 °C. The DSC spectrum of 5 (18.75% PbO content), Fig. 11B4, exhibited three endotherms at 51 °C (energy 11.01 J/g), 219 (energy 17.87 J/g), 287 sharp endotherm (energy 363.7 J/g), an endotherm at 405 C (energy 23.76 J/g), an exotherm at 480 C (energy 16.73 J/g), and an exotherm at 527 C (energy 16.73 J/g). The TGA/DrTGA profile curves of the composite containing 23.53% PbO film 5, Fig. 10B5, displayed three decomposition peaks at 209 °C (8.45 % wt loss), 347 °C (54.18 % wt loss), and 687 °C (23.80 % wt loss), leaving a 11.87 % value at 693 °C as residue. The DTG showed a weak broad exotherm at 185 °C, a strong main exotherm at 283.9 °C, and a weak endotherm at 417.3 °C. The DSC spectrum of 5 (23.53 % PbO content), Fig. 11B5, exhibited three endotherms at 187 °C (energy 4.49 J/g), 217 °C (energy 6.78 J/g), 288 sharp endotherm (energy 296.4 J/g), and an endotherm at 669 °C (energy 20.09 J/g).

Thermal studies of the composite films proved their unambiguous thermal stability. Heating the samples of films 4 containing 13.33% and 18.75 % PbO film (Fig. 10B3 and B4 up to 700 °C left 9.75 % and 17.77 % of the sample weights, respectively, as remaining metal oxide residues^19^. Interestingly, the TGA software reported an apparent final mass value of −5.16 wt% for the film containing 7.14% PbO (Fig. 10B2). Since negative residual masses are physically impossible, this value is attributed to baseline drift and instrumental correction uncertainties at high temperatures, and the actual residual mass is considered approximately zero within the experimental error. In contrast, the film containing 23.53% PbO (Fig. 10B5) retained 11.87 % of its original weight as residue at the end of the TGA run. As illustrated in Fig. 10, the thermal decomposition of all PVA/PVP-based films occurs through three degradation steps in the temperature range 207–401 °C, attributed to dehydration and degradation of other combined residuals of film ingredients^12^. Generally, for such polymeric compositions, at temperatures higher than 213 °C, the chain radical mechanism becomes relevant, and the melting of C–C or C–H bonds takes place^13^. The first degradation peak at approximately 133 °C was ascribed to the loss of contaminated moisture. The maximum degradation temperatures of the films were 401 °C, 345 °C, 329 °C, 360 °C, and 347 °C, respectively. Interestingly, the major degradation peaks of films containing PbO (Fig. 10B2–B5) were at temperatures lower than that of the neat polymeric sample 4 (Fig. 10B1). These results showed that the incorporation of different ratios of PbO into the polymeric mixture decreased the thermal resistance within the polymeric chain. The observed shifted thermal degradations can be attributed to the presence of metal oxide particles that inhibit the expected hydrogen bonding and other physical interactions between the polymeric chains and, consequently, negatively impact the thermal stability of films. Noteworthy, as reported in the literature, PbO nanoparticles were stable in a temperature range between room temperature to 1175 °C in air with the ramp rate of 10 °C min^−1^^20^.

### UV–vis characterization of composite films

**Fig. 12:**
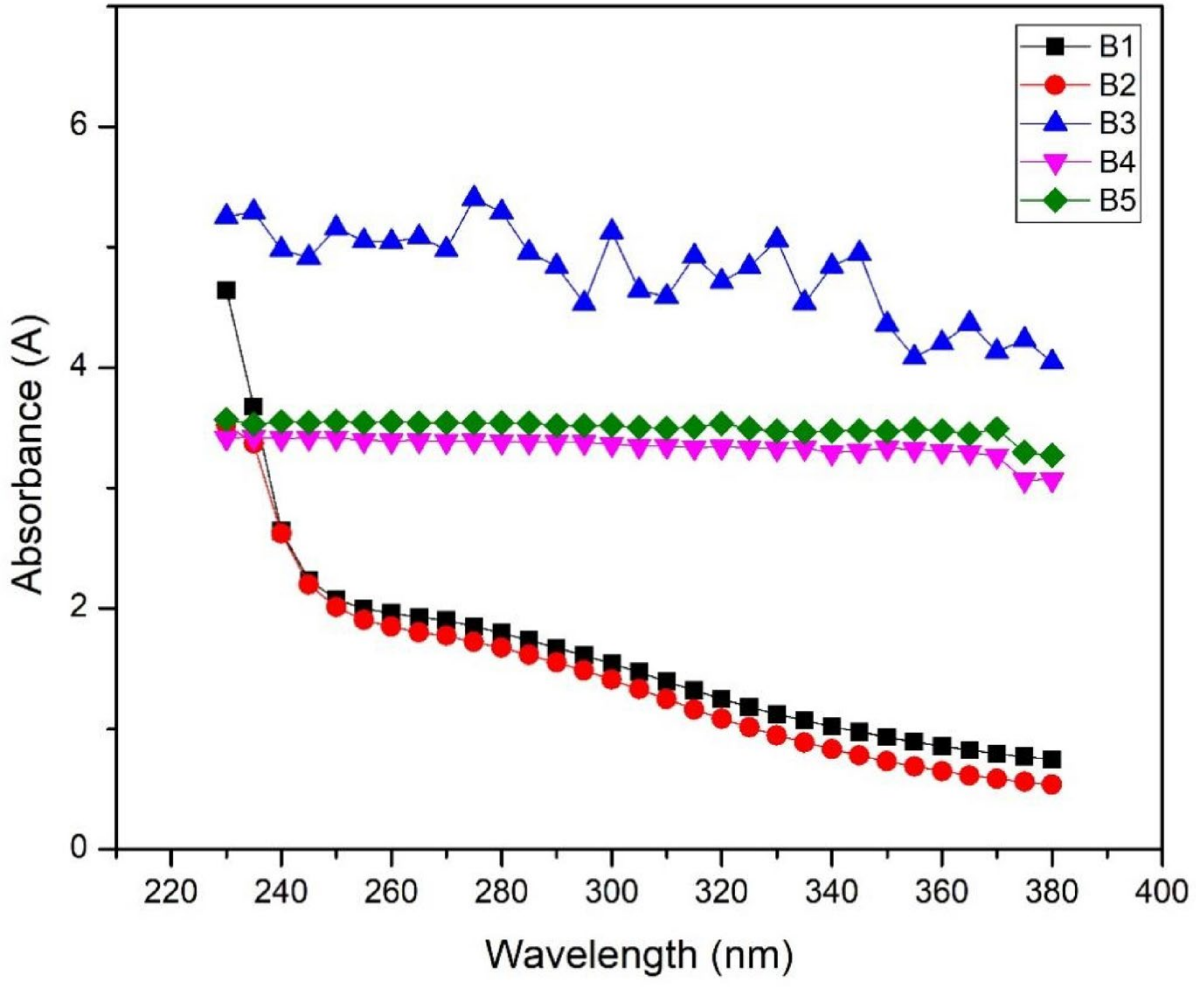
UV–vis characterization for the free polymer film (B1) and nanocomposite samples with varying additive concentrations (B2, B3, B4, B5).

Fig. 12 kind of shows the UV–vis absorbance spectra for the PVA/PVP/o-carboxymethyl chitosan/citric acid/PbO nanocomposites made with different PbO concentrations. The absorbance response is, in general, really affected by how much PbO you add, so you can see differences between the tested samples clearly. The B1 sample, that has no PbO at all, gives moderate absorbance values, and when you start adding PbO the optical absorption first goes up. In Fig. 12 it is clear that B3, with 13.33 wt.% PbO, reaches the highest absorbance across the whole wavelength area from 230 to 380 nm. This improvement is usually linked to the best dispersion of the PbO nanoparticles inside the polymer host, which means you get more active absorption spots and stronger coupling between the incoming photons and the composite skeleton. Adding PbO also brings extra electronic states that help the material take in photons, and then this supports better optical shielding. Still, when the PbO content is pushed higher than 13.33 wt.% (samples B4 and B5), the absorbance drops. One possible reason is that at higher filler levels the nanoparticles start to clump together, that kind of agglomeration reduces the real, effective surface area that photons can interact with, and it also weakens the absorption process. so, in summary, the B3 nanocomposite appears to have the best PbO amount for maximizing UV–vis absorbance, and it shows stronger optical attenuation compared to the other prepared samples.

### Water vapor permeability (WVP)

**Fig. 13:**
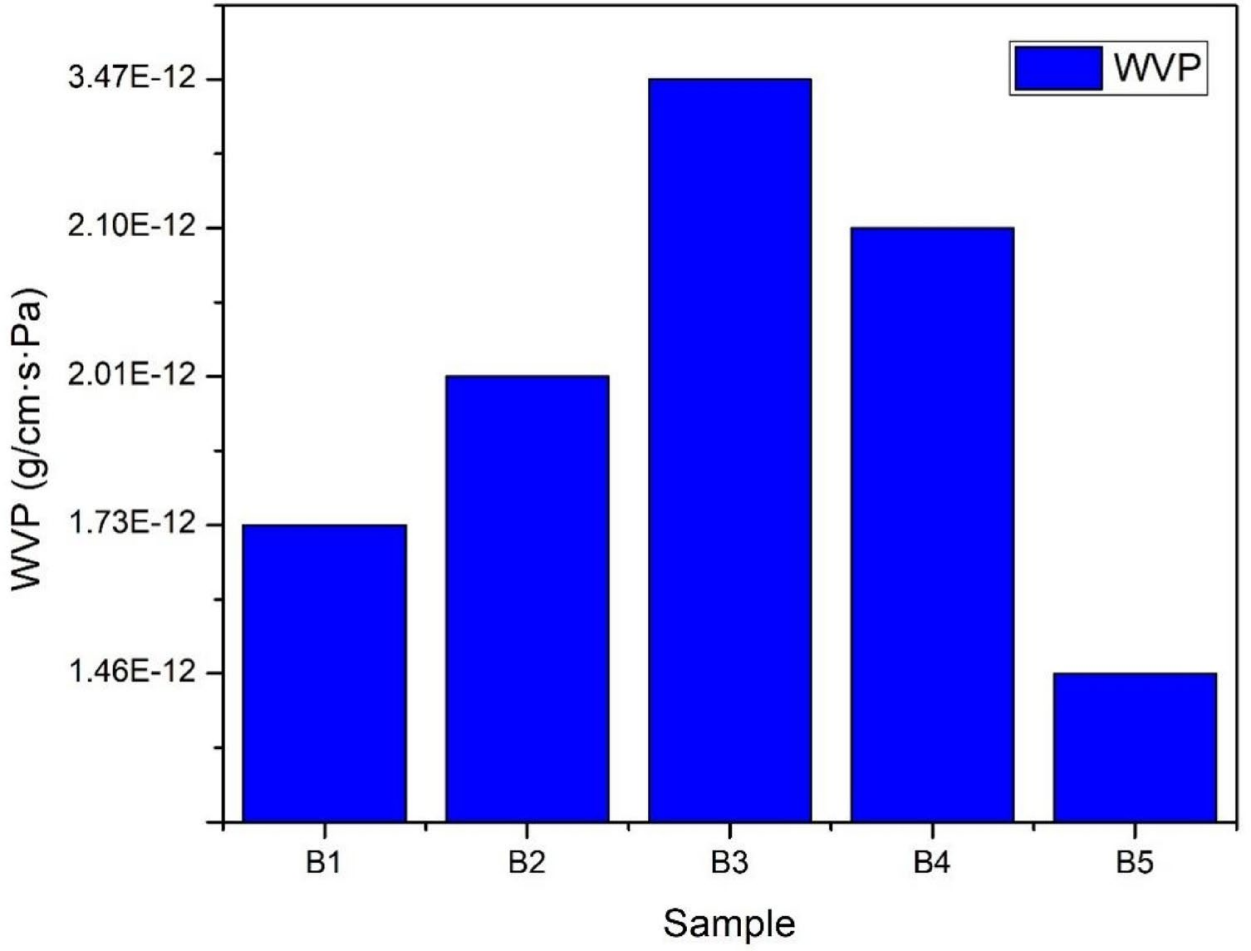
Water vapor permeability for the free polymer film (B1) and nanocomposite samples with varying additive concentrations (B2, B3, B4 and B5).

The initial formulation was followed by the incorporation of lead oxide (PbO) in four film samples at different concentrations. The corresponding WVP values were 2.01×10^−12^, 3.47×10^−12^, 2.10×10^−12^, and 1.46×10^−12^g/cm·s·Pa, respectively, as shown in Fig. 13, which are high when compared with the control film without PbO, where a WVP of 1.73×10^−12^g/cm·s·Pa was observed. These results indicate that PbO affected the water vapor barrier properties of films under concentration.

In three of the PbO-containing films, WVP values were higher than the control, which indicates that PbO at some concentrations might disturb the structural integrity of the polymer matrix. The cause could be either disruption in intermolecular hydrogen bonding or reduction of crosslink density, which could result in either more free volume or microstructural irregularities that allow more water vapor transmission. On the contrary, the film containing PbO with the lowest WVP (1.46×10^−12^g /cm·s·Pa) indicates that at an optimal concentration, PbO can actually improve the barrier properties of the structure. This improvement could be due to PbO, acting as an efficient filler, causing polymer chains to pack tighter and create less space between them. This indicates the dual role of PbO as a possible disruptor as well as an enhancer of water vapor barrier performance depending on its concentration. Hence, proper optimization of PbO amount should be extensively studied to achieve the required balance between structural integrity and barrier efficiency of the composite polymer films.

### Total soluble matter (water absorption)

**Fig. 14:**
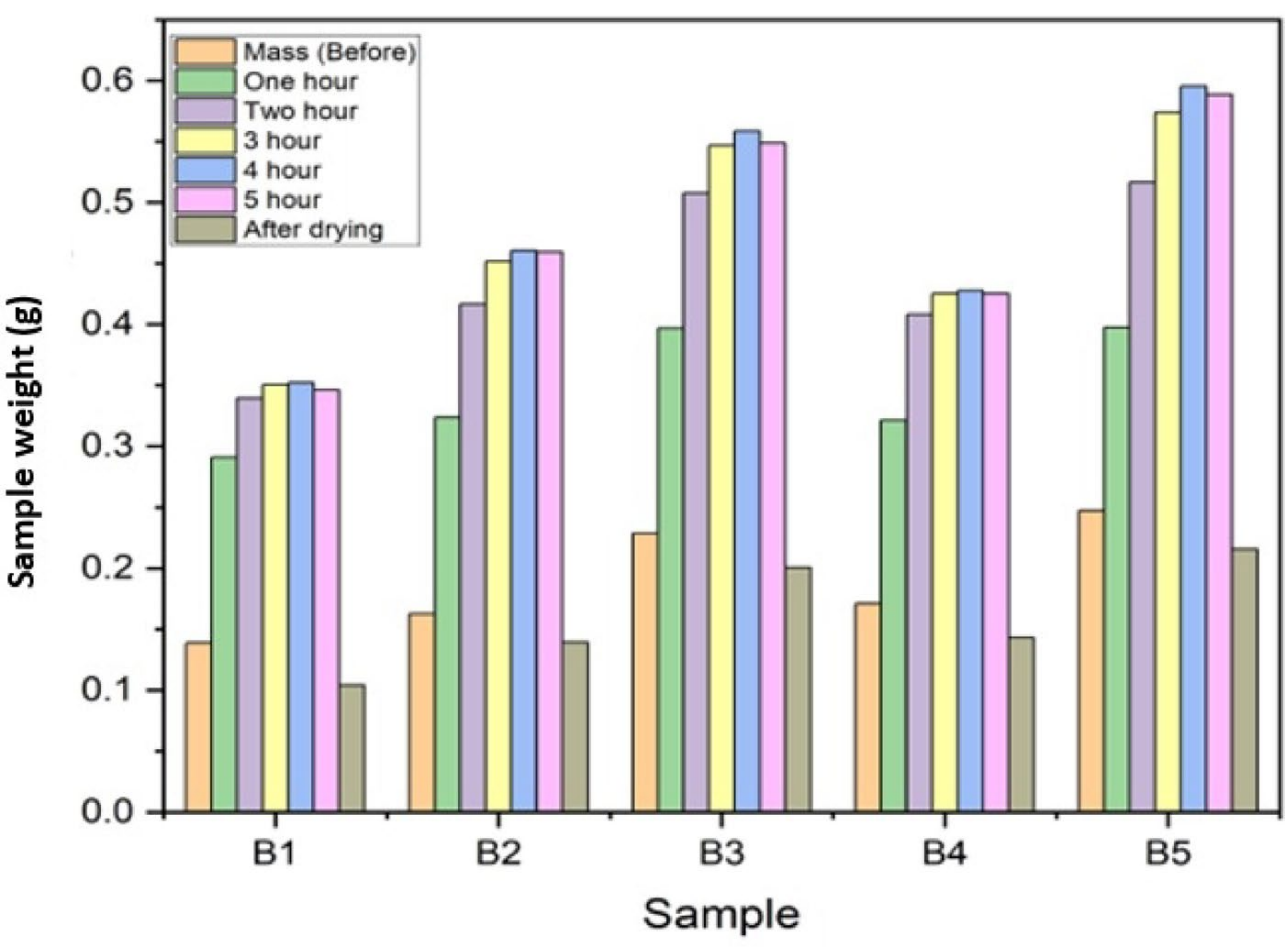
Variation in sample weight (g) for composites B1–B5 as a function of water immersion time (1–5 h) and after drying.

**Fig. 15:**
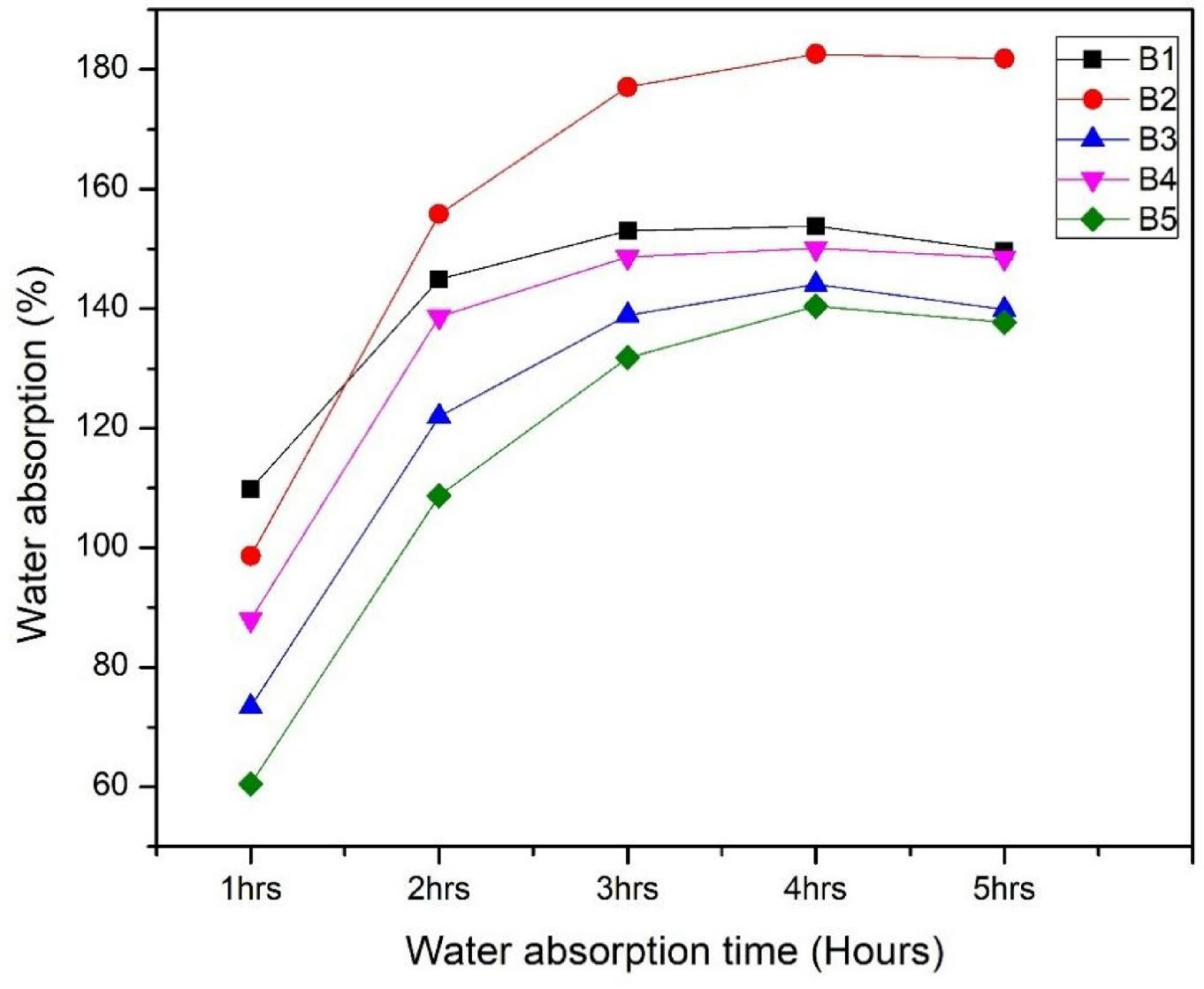
Total soluble matter (water absorption) for the free polymer film (B1) and nanocomposite samples with varying additive concentrations (B2, B3, B4, B5).

Figure 14 illustrates the variation in sample weight (g) for composites B1–B5 as a function of water immersion time (1–5 h) and after drying. It shows a steady increase in mass during immersion due to the water uptake and a considerable decrease after drying, thereby proving the reversibility of water absorption and retention behavior for each composite formulation. Water absorption behavior for the prepared composites (B1–B5) is shown in Fig. 15. All the samples demonstrated a rapid increase in water uptake during the first 2–3 h and gradual approach to saturation thereafter. Among the various formulations, B2 showed the maximum absorption of water (≈180%), indicating its higher hydrophilic nature or enhanced porosity compared to the other samples. B1, even though, showed comparatively higher absorption but remained slightly less than B2. On the contrary, B5 had the least uptake of water during the entire immersion time, which corroborates the composition working well, probably due to the inclusion of nano PbO and crosslinking agents for reducing the number of available pores and enhancing the water resistance. For B3 and B4, moderate absorption points toward partial enhancement of water barrier properties. in brief, the trends indicate that both increasing filler content and crosslinking density effectively inhibit water absorption and further moisture resistance of the composites.

### Soil burial degradation

The results revealed a remarkable loss in the original weight of the chitosan-containing plastic film samples for six months while in soil, with mass loss percentages ranging from 8.4 to 16.1 (Table 2). This loss can be attributed to partial biodegradation in natural environmental conditions, primarily due to chitosan’s presence in the film matrix. Chitosan is a naturally occurring polysaccharide derived from chitin that is very easy to biodegrade by microbes and enzyme activity, particularly by soil microorganisms like fungi and bacteria. These organisms possess the ability to produce their enzymes—namely chitinases and chitosanases that hydrolyze the glycosidic bonds within the chitosan backbone. Also, having added chitosan into the plastic matrix will change its physical and chemical properties towards a more hydrophilic and oxygen-permeable nature, encouraging microbial colonization and then enzymatic degradation. Variability in mass loss across samples may be due to differences in film thickness, local microenvironmental conditions, or heterogeneity in microbial activity within the soil. In summary, the findings would indicate that the inclusion of chitosan in plastic films could enhance biodegradation, as it is an encouraging approach for developing eco-friendly materials with less long-term ecological impact^21^.

#### Gamma-ray-shielding characteristics

The mass attenuation coefficient (MAC) represents a crucial criterion for assessing the radiation shielding capability of the plastic film under consideration. Hence, the mass attenuation coefficient (MAC) was determined experimentally using standard point sources that emit gamma rays with energies ranging from 0.0595 MeV to 1.3325 MeV. The experimental values of MAC were therefore compared with theoretical values obtained from the XCOM database. Both results, along with the computed deviation between experimental and theoretical values, are presented in Table 3. The percentage deviation was calculated using the following equation.

10 $${\mathrm{Deviation}} \left( \% \right) = \frac{{\left| {MAC_{XCOM} - MAC_{EXP} } \right|}}{{MAC_{XCOM} }} \times 100$$

**Table 3:** Comparison of experimental MAC values for the free polymer film and nanocomposite samples with varying additive concentrations.

Sample	Energy (MeV)	Density (g·cm^−3^)	MAC		
			EXP.	XCOM	Deviation %
B1	0.0595	1.250	0.1822±0.0011	0.1832	0.553
	0.0810		0.1689±0.0023	0.169	0.0769
	0.1218		0.1508±0.0005	0.1526	1.1526
	0.3560		0.1077±0.0001	0.1079	0.2235
	0.6617		0.0832±0.0041	0.0834	0.2804
	1.1732		0.0635±0.0011	0.0636	0.1404
	1.3325		0.0588±0.0007	0.0596	1.2867
B2	0.0595	1.333	0.5303±0.0015	0.4780	10.9395
	0.0810		0.3213±0.0003	0.2933	9.5457
	0.1218		0.3829±0.0022	0.3566	7.3721
	0.3560		0.1247±0.0008	0.1183	5.4125
	0.6617		0.0885±0.0004	0.0847	4.4339
	1.1732		0.0650±0.0013	0.0633	2.7148
	1.3325		0.0607±0.0011	0.0592	2.4335
B3	0.0595	1.414	0.8203±0.0014	0.7335	11.8288
	0.0810		0.4431±0.0004	0.4010	10.5026
	0.1218		0.5777±0.0013	0.5334	8.2989
	0.3560		0.1373±0.0041	0.1273	7.8883
	0.6617		0.0902±0.0017	0.0859	5.0427
	1.1732		0.0660±0.0001	0.0631	4.7338
	1.3325		0.0614±0.0004	0.0589	4.2827
B4	0.0595	1.494	1.0876±0.0020	0.9571	13.6313
	0.0810		0.5531±0.0018	0.4953	11.6610
	0.1218		0.7577±0.0015	0.6880	10.1289
	0.3560		0.1471±0.0003	0.1352	8.8039
	0.6617		0.0935±0.0003	0.0869	7.6230
	1.1732		0.0668±0.0022	0.0628	6.2457
	1.3325		0.0617±0.0014	0.0586	5.2628
B5	0.0595	1.572	1.3209±0.0014	1.1540	14.4586
	0.0810		0.6519±0.0008	0.5785	12.6951
	0.1218		0.9154±0.0014	0.8245	11.0210
	0.3560		0.1552±0.0032	0.1422	9.1175
	0.6617		0.0950±0.0061	0.0877	8.3241
	1.1732		0.0669±0.0053	0.0626	6.7947
	1.3325		0.0617±0.0004	0.0584	5.6892

The results in Table 3, well they show this pretty clear strong dependence on photon energy and atomic number (Z) and density, plus the whole material microstructure and overall, the data keep showing this steady kind of drop in MAC as photon energy goes up, for all the samples B1–B5. Like, MAC goes down from the low–energy area 0.0595 MeV to the high–energy area 1.3325 MeV, which kind of lines up with the change in what interaction dominates: at low energies it’s mostly photoelectric absorption, then around intermediate energies it shifts toward Compton scattering, and then at higher energies there’s a bit of pair production (>1.022 MeV) in the mix^22–24^. So that overall inverse relationship between MAC and energy comes out, pretty much as expected; for instance in the most loaded nanocomposite B5, MAC drops from 1.3209 cm^2^/g at 0.0595 MeV to 0.0617 cm^2^/g at 1.3325 MeV, and for B1 through B4 you still see basically the same descending pattern, so this kind of “universal” energy dependence gets confirmed.

On the atomic number and density side, Table 3 also gives a systematic story: MAC rises when the filler content and density rise, starting at B1 (ρ = 1.250 g·cm⁻^3^) and reaching B5 (ρ = 1.572 g·cm⁻^3^). Here, the extra PbO loading makes the composite’s effective atomic number and electron density higher, so attenuation improves a lot, especially at low photon energies where the photoelectric effect is the main player. You can see that clearly in the 0.0595 MeV case: MAC jumps from 0.1822 cm^2^/g for B1 to 1.3209 cm^2^/g for B5. This is a strong sign that the high-Z PbO matters a lot for strengthening photon absorption; at intermediate energies, say 0.3560 and 0.6617 MeV, the sample to sample gap becomes less obvious, which fits with Compton scattering being more about electron density rather than atomic number, even though the higher-Z specimens still usually give better shielding.

For validation against theory, the XCOM values are treated as the bulk theoretical baseline, and the comparison indicates experimental MAC for the nanocomposites (B2–B5) are consistently higher than XCOM, with the positive deviation increasing as filler concentration increases, roughly in the 2–14% range. That suggests the nano-structuring is improving efficiency beyond what bulk expectations predict. This is plausibly tied to improved dispersion of PbO nanoparticles, less agglomeration, a larger interfacial area, and a more uniform electron density spread, all of which increase the chance of photon interaction. Meanwhile, the plain polymer film (B1) tracks XCOM more closely, so it behaves more like a bulk-like low-Z matrix. In the end, Table 3 basically confirms: MAC increases with atomic number and density, decreases with photon energy, and is noticeably boosted in the PbO nanocomposites vs the bulk theoretical predictions. That points to nano-scale incorporation helping radiation shielding mainly through microstructural refinement, not because the fundamental photon interaction mechanisms themselves have fundamentally changed.

**Fig. 16:**
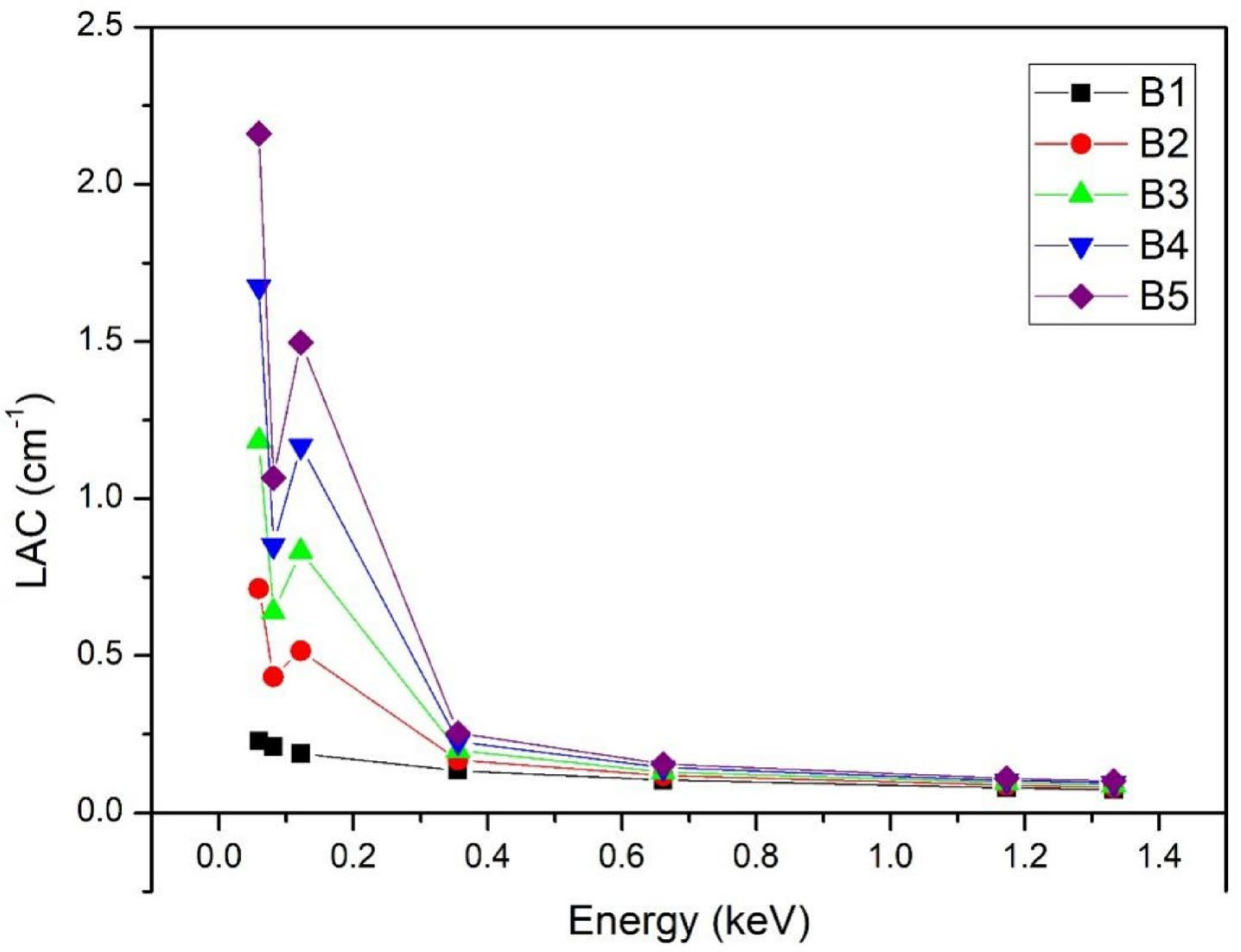
Linear attenuation coefficients for the free polymer film (B1) and nanocomposite samples with varying additive concentrations (B2, B3, B4, B5).

Using narrow-beam gamma-ray transmission geometry, the linear attenuation coefficients were measured for the samples under study. The result is shown in Fig. 16. The measured values of LAC for materials of different concentrations of PbO nanoparticles were directly dependent on the density of the materials, which increased proportionally with PbO content due to the high atomic number and mass density of lead. As can be seen from the results, as the PbO concentration increases, the interaction probability of photons within the medium increases, enhancing the shielding capability. Moreover, the LAC had a typical decreasing trend with increasing photon energy due to the penetration abilities of high-energy photons and their reduced interaction cross-section. Furthermore, a sudden increase in the photon attenuation coefficient was observed at 121.8 keV, which can be attributed to the presence of the K-absorption edge of Pb occurring at 88 keV. Further, some small changes in attenuation values are caused by differences in the structure of the nanomaterial form of PbO, which can change the trajectory of the photons and thus marginally increase interaction probability on a nanoscale.

**Fig. 17:**
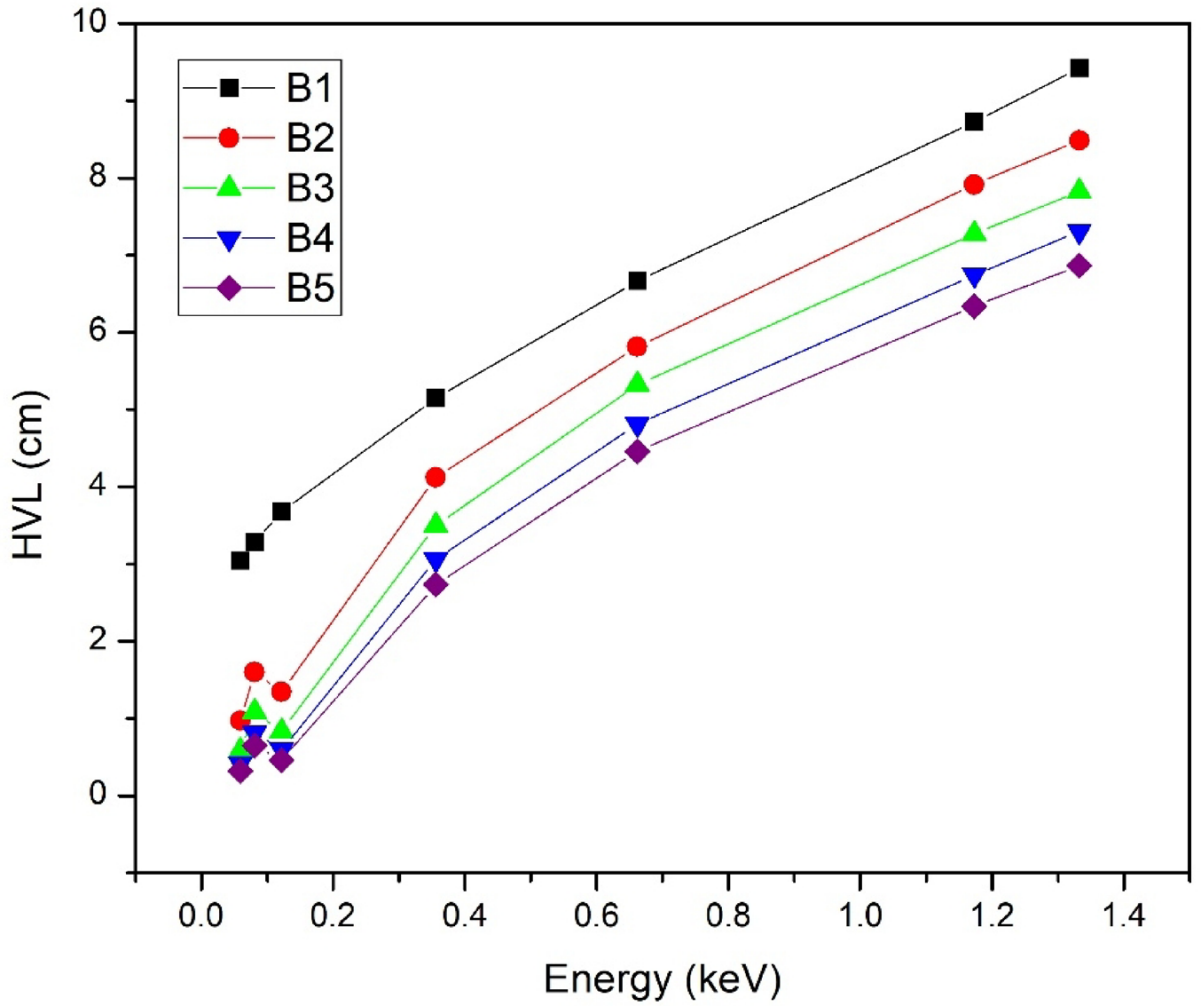
The variation of HVL as a function of photon energy for the free polymer film (B1) and nanocomposite samples with varying additive concentrations (B2, B3, B4, B5).

**Fig. 18:**
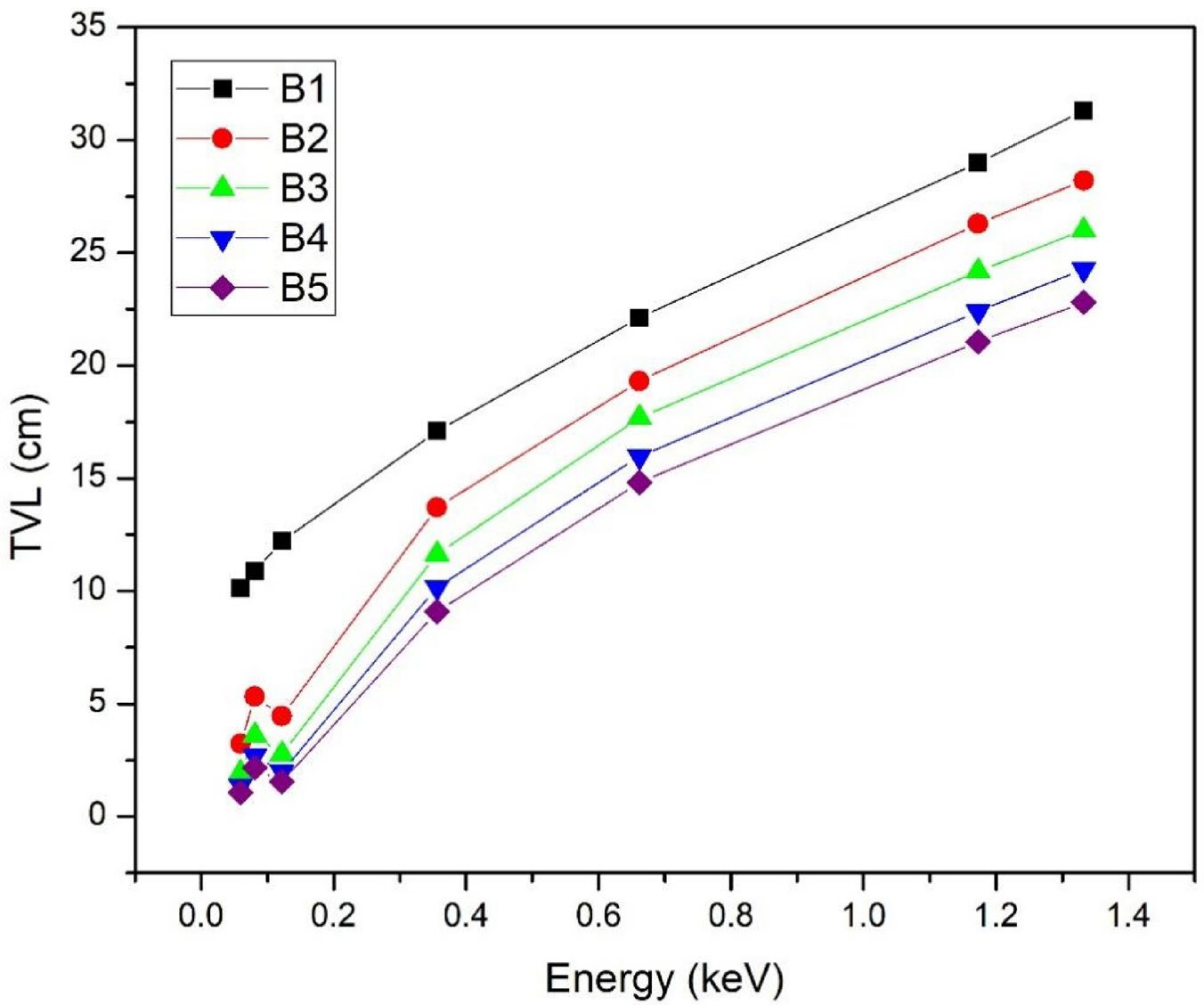
The variation of TVL as a function of photon energy for the free polymer film (B1) and nanocomposite samples with varying additive concentrations (B2, B3, B4, B5).

**Fig. 19:**
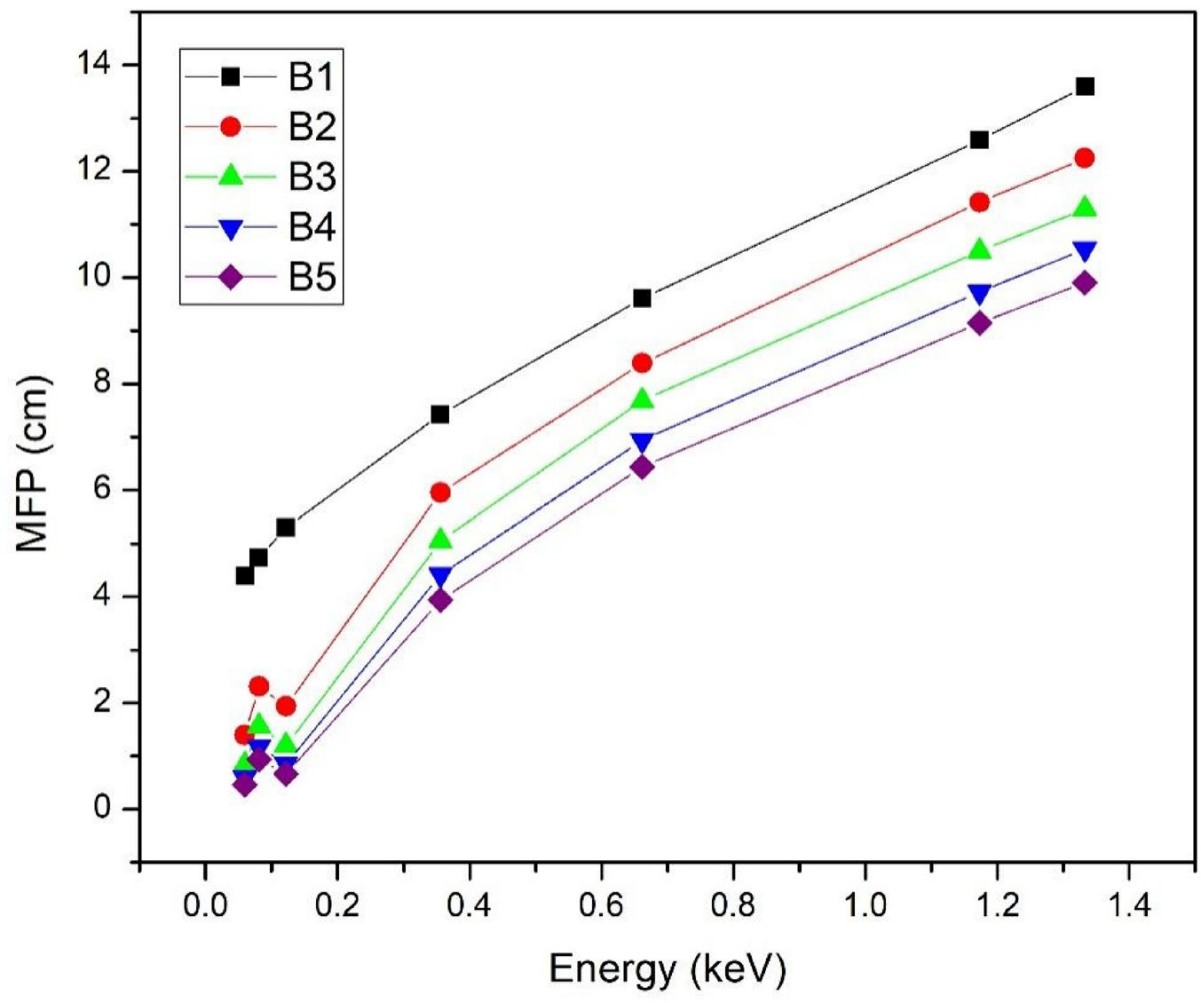
The variation of MFP as a function of photon energy for the free polymer film (B1) and nanocomposite samples with varying additive concentrations (B2, B3, B4, B5).

The performance evaluation of the manufactured plastic-based shielding materials is carried out by determining important radiation attenuation parameters, viz., half-value layer (HVL), tenth-value layer (TVL), and mean free path (MFP). These parameters are predominantly used to evaluate the capacity of any material to shield against gamma radiation. HVL and TVL are defined as the thicknesses of the material required to lower the incident gamma-ray intensity to 50% and 10%, respectively, of its original value. The MFP denotes the average distance a photon travels in the material before its interaction. Small HVL, TVL, and MFP values denote better shielding capabilities. Figures 17, 18, and 19 display how the HVL, TVL, and MFP change with photon energy for all the prepared plastic samples. In general, all three parameters increase with increasing photon energy, indicating that these higher-energy photons can penetrate the shielding material more efficiently. An exception was observed at 121.78 keV, where a sudden decrease in the HVL, TVL, and MPF values occurred for all PbO-reinforced samples, attributable to the K-absorption edge of Pb at 88 keV. Also, a significant drop in HVL, TVL, and MFP was observed for all the samples reinforced with PbO, with increasing concentration, proving a remarkable improvement in radiation attenuation.

Table 4 shows how the linear attenuation coefficients (μ) for the prepared PVA/PVP/CMCN/citric acid/PbO nanocomposite (B5) compare with some earlier shielding materials, at photon energies 0.6617 MeV (^137^Cs) and 1.1732 MeV (^60^Co). In Table 4 it is apparent that μ tends to go down when photon energy goes up, for all of the studied materials. This happens because the chance of photon interaction becomes less at higher energy, so the radiation just passes more easily. For the B5 nanocomposite, which includes 23.53 wt.% PbO, the linear attenuation coefficients are 0.1493 and 0.1051 cm⁻^1^ at 0.6617 MeV and 1.1732 MeV, respectively. These numbers are noticeably larger than the coefficients reported for polyaniline, the unsaturated polyester/nanoclay/30% PbO composite, and for Poly (HEMA-co-Styrene)/30% WO₃·2H₂O at the same energies. Overall, that suggests a stronger gamma shielding behavior of the made material. Even so, barite concrete and ordinary concrete are still commonly chosen shielding options, mainly because of their high density and stable structural characteristics. Still, the B5 polymer composite manages to give competitive attenuation performance, while also bringing extra perks like less weight and easier processing. The better attenuation ability of B5 is likely linked to PbO. Lead has a high atomic number, and those Pb atoms help a lot with photon absorption and scattering effects. So, putting it together, the findings in Table 4 basically support that the PbO-reinforced polymer nanocomposite prepared here could be a suitable and promising candidate for gamma-radiation shielding uses.

**Table 4:** Comparison study of linear attenuation coefficients (cm^-1^) with literature.

Composites	Linear attenuation coefficients μ (cm^-1^)		References
	^137^Cs (0.6617 MeV)	^60^Co (1.1732 MeV)	
Barite concrete	0.172	0.127	Mahmoud et al.^25^
Ordinary concrete	0.181	–	Sharifi et al.^26^
Polyaniline	0.111	0.084	Kaçal et al.^27^
Unsaturated polyester/nano clay/30% PbO	0.133	0.049	Bagheri et al.^28^
Poly(HEMA-co- Styrene)/%30 WO3.2H20 Composite	0.135	0.096	Körpınar et al.^29^
VA/PVP/CMCN/Citric acid/23.53 wt.% PbO (B5)	0.1493	0.1051	Present work

## Environmental and safety considerations

We note that PbO is toxic and that its inclusion in polymer films raises potential environmental and health concerns, particularly if particles can leach or become airborne. In our composites the PbO is embedded in a crosslinked PVA/PVP/O-carboxymethyl chitosan matrix, which can reduce particle mobility by physical encapsulation and limit immediate release. However, embedding alone does not guarantee long-term containment; targeted analyses such as standardized leaching tests (TCLP or simulated rainwater leaching), accelerated aging and abrasion tests, and long-term soil/water migration studies are required to quantify release risks. To mitigate hazards in practical applications we recommend (i) evaluating encapsulation strategies or surface coatings to prevent wear and particle release, (ii) exploring less-toxic high-Z fillers (for example Bi_2_O_3_, WO_3_, or BaSO_4_) as potential alternatives, and (iii) planning end-of-life protocols for collection and hazardous-waste disposal or recycling. These steps would be necessary before proposing the materials for real-world use where Pb exposure or environmental release is a concern

## Conclusion

This work successfully synthesized carboxymethyl chitosan /PbO nanocomposite films using the solution casting method, representing a promising approach for the development of radiation shielding materials**.** The incorporation of PbO nanoparticles into the chitosan matrix produced compact films with nanoparticles uniformly dispersed throughout the polymer framework. This structural uniformity was crucial in improving the shielding performance of the composites. Experimental analysis demonstrated that the nanocomposite films exhibited excellent gamma-ray attenuation properties, as confirmed by their linear and mass attenuation coefficients. These values were in close agreement with theoretical predictions from XCOM, reinforcing the accuracy and reliability of the developed materials. Moreover, shielding parameters such as the half-value layer (HVL), tenth-value layer (TVL), and mean free path (MFP) highlighted the strong ability of the nanocomposites to reduce gamma radiation over a wide energy range of 59.5–1332 keV. Such findings point to their suitability in environments with varying intensities of gamma exposure. Beyond shielding, the nanocomposites displayed outstanding physicochemical properties. Characterization through SEM, TEM, EDX, and XRD confirmed their structural integrity, homogeneous nanoparticle dispersion, and crystalline features, while UV–Vis analysis revealed their optical behavior. The partial biodegradability of the polymer matrix was further shown by water vapor permeability (WVP) and soil burial experiments; nevertheless, more research is needed to evaluate the possible safety and environmental effects of PbO nanoparticle presence. Overall, the work shows that chitosan/PbO nanocomposite films combine biodegradable polymer properties with efficient gamma radiation shielding. These composites show promise for radiation shielding applications, but before they are widely used in practice, their safety and environmental constraints need to be further assessed. To fully establish their real-world applicability, future efforts should focus on scaling up production, optimizing nanoparticle concentrations, and evaluating them under real-world conditions. This should include testing for leaching and nanoparticle release, abrasion resistance, a detailed examination of mechanical properties, and life-cycle or end-of-life disposal evaluations.

## Data Availability

The datasets used and/or analyzed during the current study are available from the corresponding author on reasonable request.
